# Role of antiseizure medication in postoperative late seizure and survival among patients with newly diagnosed glioblastoma: A multicenter retrospective study

**DOI:** 10.1093/noajnl/vdag205

**Published:** 2026-08-07

**Authors:** Akihiro Inoue, Makoto Ohno, Yawara Nakamura, Yoshitaka Narita, Daisuke Kawauchi, Yoshiteru Shimoda, Mitsuto Hanihara, Tomoo Matsutani, Shigeo Ohba, Natsumi Yamashita, Narushi Sugii, Kuniaki Saito, Ichiyo Shibahara, Shunsaku Takayanagi, Joji Ishida, Masamichi Takahashi, Noriyuki Kijima, Yuta Mitobe, Nayuta Higa, Masato Saito, Ryosuke Matsuda, Yukitomo Ishi, Kohei Nakajima, Shinji Yamashita, Kaoru Tamura, Iori Ozono, Taiki Setoguchi, Tetsuya Negoto, Kosuke Katayama, Atsushi Kambe, Daisuke Kuga, Kazuhiro Tanaka, Takeshi Okuda, Koichiro Sumi, Takahiro Kanda, Shigeki Takada, Kojiro Wada, Yu Fujii, Yu Kawanishi, Hideyuki Arita, Yuichi Sato, Takuya Yonemochi, Ryohei Otani, Shinichiro Koizumi, Yoshiki Arakawa, Takeharu Kunieda

**Affiliations:** Department of Neurosurgery, Ehime University Graduate School of Medicine, Toon, Ehime, Japan; Department of Neurosurgery and Neuro-Oncology, National Cancer Center Hospital, Chuo-ku, Tokyo, Japan; Department of Neurosurgery, Ehime University Graduate School of Medicine, Toon, Ehime, Japan; Department of Neurosurgery and Neuro-Oncology, National Cancer Center Hospital, Chuo-ku, Tokyo, Japan; Department of Neurosurgery and Neuro-Oncology, National Cancer Center Hospital, Chuo-ku, Tokyo, Japan; Department of Neurosurgery and Neuro-Oncology, National Cancer Center Hospital, Chuo-ku, Tokyo, Japan; Department of Neurosurgery, Tohoku University Graduate School of Medicine, Sendai, Miyagi, Japan; Department of Neurosurgery, Interdisciplinary Graduate School of Medicine and Engineering, University of Yamanashi, Chuo, Yamanashi, Japan; Department of Neurological Surgery, Graduate School of Medicine, Chiba University, Chiba, Chiba, Japan; Department of Neurosurgery, Fujita Health University School of Medicine, Toyoake, Aichi, Japan; Clinical Research Center, NHO Shikoku Cancer Center, Matsuyama City, Ehime, Japan; Clinical Therapeutic Trial Center, Ehime University Hospital, Toon City, Ehime, Japan; Department of Neurosurgery, Institute of Medicine, University of Tsukuba, Tsukuba, Ibaraki, Japan; Department of Neurosurgery, Kyorin University School of Medicine, Mitaka, Tokyo, Japan; Department of Neurosurgery, Kitasato University School of Medicine, Sagamihara, Kanagawa, Japan; Department of Neuro-Oncology, International Medical Center, Saitama Medical University, Hidaka-city, Saitama, Japan; Department of Neurological Surgery, Okayama University Graduate School of Medicine, Dentistry, and Pharmaceutical Sciences, Okayama, Japan; Department of Neurosurgery and Neuro-Oncology, National Cancer Center Hospital, Chuo-ku, Tokyo, Japan; Department of Neurosurgery, Tokai University School of Medicine, Isehara, Kanagawa, Japan; Department of Neurosurgery, The University of Osaka Graduate School of Medicine, Suita, Osaka, Japan; Department of Neurosurgery, School of Medicine, Yamagata University, Yamagata, Japan; Department of Neurosurgery, Kagoshima University Graduate School of Medical and Dental Sciences, Kagoshima City, Kagoshima, Japan; Department of Neurosurgery, Asahikawa Medical University, Asahikawa City, Hokkaido, Japan; Department of Neurosurgery, Nara Medical University, Kashihara, Nara, Japan; Department of Neurosurgery, Hokkaido University School of Medicine, Sapporo, Japan; Department of Neurosurgery, Tokushima University Hospital, Tokushiuma, Japan; Division of Neurosurgery, Department of Clinical Neuroscience, Faculty of Medicine, University of Miyazaki, Miyazaki City, Miyazaki, Japan; Department of Neurosurgery, Institute of Science Tokyo, Bunkyo-ku, Tokyo, Japan; Department of Neurosurgery, Graduate School of Biomedical and Health Sciences, Hiroshima University, Hiroshima City, Hiroshima, Japan; Department of Neurosurgery, Chiba Cancer Center, Chiba City, Chiba, Japan; Department of Neurosurgery, Kurume University School of Medicine, Kurume, Fukuoka, Japan; Department of Neurosurgery, Hirosaki University Graduate School of Medicine, Hirosaki-shi, Aomori, Japan; Division of Neurosurgery, Department of Brain and Neurosciences, Faculty of Medicine, Tottori University, Yonago, Tottori, Japan; Department of Neurosurgery, Graduate School of Medical Sciences, Kyushu University, Fukuoka, Japan; Department of Neurosurgery, Kobe University Graduate School of Medicine, Kobe, Japan; Department of Neurosurgery, Faculty of Medicine, Kindai University, Sakai, Osaka, Japan; Department of Neurological Surgery, Nihon University School of Medicine, Itabashi-ku, Tokyo, Japan; Department of Neurological Surgery, Faculty of Medicine, Kagawa University, Kita-gun, Kagawa, Japan; Department of Neurosurgery, Kyoto University Graduate School of Medicine, Kyoto, Japan; Department of Neurosurgery, National Defense Medical College, Tokorozawa, Saitama, Japan; Department of Neurosurgery, Shinshu University School of Medicine, Matsumoto, Nagano, Japan; Department of Neurosurgery, Kochi Medical School, Kochi University, Nankoku, Kochi, Japan; Department of Neurosurgery, Osaka International Cancer Institute, Osaka, Japan; Department of Neurosurgery, Iwate Medical University, Shiwa, Iwate, Japan; Department of Neurosurgery, Tokai University School of Medicine, Isehara, Kanagawa, Japan; Department of Neurosurgery, Tokyo Metropolitan Cancer and Infectious Diseases Center Komagome Hospital, Bunkyo-ku, Tokyo, Japan; Department of Neurosurgery, Hamamatsu University School of Medicine, Hamamatsu, Shizuoka, Japan; Department of Neurosurgery, Kyoto University Graduate School of Medicine, Kyoto, Japan; Department of Neurosurgery, Ehime University Graduate School of Medicine, Toon, Ehime, Japan

**Keywords:** antiseizure medication, brain tumor-related epilepsy, glioblastoma, glutamate, seizure suppression

## Abstract

**Background:**

We conducted a multicenter study to investigate the utility of postoperative antiseizure medication (ASM) in the occurrence of late seizure in glioblastoma. In addition, we analyzed whether postoperative ASM affects survival.

**Methods:**

1132 consecutive patients with newly diagnosed glioblastoma at 39 centers were enrolled. In patients treated with postoperative ASM (levetiracetam [LEV], lacosamide [LCM], or perampanel [PER]), as well as in those who received no medication, the cumulative incidence of late seizure, adverse events, and survival outcomes were analyzed.

**Results:**

In total, 1099 patients (female: 482, male: 617, mean age: 67.2 years) were included, among whom, 287 developed postoperative late seizure. The mean onset time of late seizure was 193.6 days after surgery. Postoperative ASMs were administered in 628 cases, LEV in 344, LCM in 124, and PER in 160. The cumulative incidence of late seizure at 12 months was as follows: no medication in 30.9%, LEV in 17.8%, LCM in 28.8%, and PER in 16.2%. Multivariable analysis disclosed that LEV and PER displayed significantly lower cumulative incidences of postoperative late seizure compared with no medication and LCM. By contrast, median progression-free survival/overall survival did not significantly differ among ASMs. However, during the 12–24 month interval, the PER group demonstrated a more favorable prognosis than the other groups.

**Conclusions:**

LEV and PER after glioblastoma surgery were associated with a reduced risk of late seizure compared with LCM or no medication. LEV or PER may be considered as postoperative ASMs to optimize seizure control and potentially help preserve patients’ QOL.

Key Points

- LEV and PER were associated with a lower incidence of late seizure compared with LCM or no medication following glioblastoma surgery.
- LEV or PER may be considered as postoperative ASM options for seizure control and QOL preservation.

Importance of the Study

Postoperative late seizures in glioblastoma (GBM) are often refractory and compromise quality of life (QOL), but the pathophysiology and effective treatment remain unclear. Therefore, we conducted a multicenter study to evaluate the impact of postoperative antiseizure medication (ASM) on late seizure control and survival in GBM. We retrospectively analyzed the cumulative incidence of late seizure and survival outcomes in patients treated with postoperative ASM (levetiracetam [LEV], lacosamide [LCM], or perampanel [PER]), as well as in those who received no medication. Our results suggested that LEV and PER after GBM surgery were associated with a lower incidence of late seizure compared with LCM or no medication. These findings suggest that LEV or PER may be considered postoperative ASMs for seizure control and QOL preservation, supporting the rational selection of postoperative ASMs with an emphasis on seizure control and QOL preservation in routine GBM management.

Epileptic seizure is a major symptom associated with malignant gliomas and significantly affects not only patients’ overall physical condition but also their quality of life (QOL). In particular, brain tumor-related epilepsy (BTRE) is classified into 2 seizure types.^1^ One is a preoperative seizure as a presenting symptom of the tumor. The other is a postoperative seizure, which is divided into early seizure (distinguished as an acute symptomatic seizure) occurring within 7 days after surgery, and late seizure developing 8 days or more after surgery. Among these seizures, preoperative seizure is caused by the presence of the tumor at onset, and postoperative early seizure may be caused by residual tumor and/or systemic diseases such as hepatic and renal dysfunction and anemia. By contrast, postoperative late seizure is regarded as a sign of tumor progression or recurrence and often displays treatment-refractory epilepsy, resulting in unfavorable outcomes in patients with glioblastoma (GBM).^1^^,^^2^ In GBM, the incidence of preoperative seizures in Japan is 14%, with a lifetime incidence of seizure of 33%.^3^ Prolonged seizures can lead to hypoxic encephalopathy, increased intracranial pressure, and irreversible neurological damage. Therefore, effective seizure control is important in the preoperative management of patients with GBM.^4^ However, details of the pathogenesis of and optimum treatment for postoperative seizure remain poorly understood. Particularly, postoperative late seizure not only causes refractory epilepsy but also may contribute to shorter survival in patients with GBM compared with preoperative seizure and postoperative early seizure.^5^

Patients with seizures have traditionally been managed with the administration of valproic acid, phenytoin, and carbamazepine. In recent years, however, newer antiseizure medications (ASMs), including levetiracetam (LEV), lacosamide (LCM), and perampanel (PER), have been introduced into clinical practice. The latest Japanese clinical practice guidelines for epilepsy, published in 2018, do not recommend these newer ASMs as first-line agents.^6^ By contrast, expert consensus in the United States indicates that many specialists had already adopted newer ASMs as first-line therapy by 2016.^7^ In Japan, LEV, LCM, and PER have become increasingly utilized because of their favorable efficacy and tolerability. Nevertheless, the choice and duration of ASMs in patients with GBM vary widely among institutions. Moreover, real‑world patterns of ASM use and the specific effects of each agent on BTRE suppression remain poorly characterized.

The present multicenter retrospective analysis aimed to clarify the most effective ASM and the optimum administration method for controlling postoperative late seizure in GBM. Data on patients who received continuous monotherapy with any of LEV, LCM, or PER for a defined postoperative period, or who did not receive ASMs, were collected from multiple institutions. In addition, to evaluate the potential antitumor properties of ASMs, we examined their associations with progression-free survival (PFS) and overall survival (OS).

## Materials and Methods

### Ethics Statement

All procedures performed in studies involving human participants were conducted in accordance with the ethical standards of the institutional and/or national research committee and with the 1964 Declaration of Helsinki and its later amendments or comparable ethical standards. This study was approved by the Ethics Committee for Clinical Research at Ehime University Hospital (approval No. 2411010).

### Patients and Study Design

Patients with GBM (IDH wild-type, central nervous system [CNS] World Health Organization [WHO] Grade 4) aged ≥ 18 years who underwent initial surgical treatment in Japan between January 2021 and December 2023 were included in this retrospective study. Histopathological diagnosis confirmed IDH wild-type GBM in all cases, according to the 2021 WHO classification of tumors of the CNS.^8^  The extent of tumor resection (EOR) was not included in the inclusion criteria; however, all cases received postoperative chemoradiotherapy based on the Stupp regimen.^9^ The EOR was categorized as gross total resection (GTR; 100% resection), non-GTR (60%-99%), or biopsy (< 60%).^10^ Biopsy was performed to establish a definitive diagnosis and guide subsequent treatment. Preoperative neurological status and functional independence were assessed using the Karnofsky Performance Status (KPS).^11^ Adverse events associated with ASMs were evaluated using the Common Terminology Criteria for Adverse Events (version 5.0), and events of Grade 3 or higher were extracted. Tumors were confined to the supratentorial region (frontal, parietal, temporal, or occipital lobes, or the insula) on magnetic resonance imaging performed immediately before surgery. Eligible patients included those who had initiated or continued single-agent ASM therapy with LEV, LCM, or PER between postoperative day 8 and 2 weeks after surgery. Prespecified treatment groups for the primary analysis were based on ASM use from postoperative day 8 to day 14. Patients without ASM use during this period were classified as the “no medication” group, regardless of subsequent ASM initiation on or after postoperative day 15, as were those who started ASM only after experiencing late seizures.

**Figure 1.**
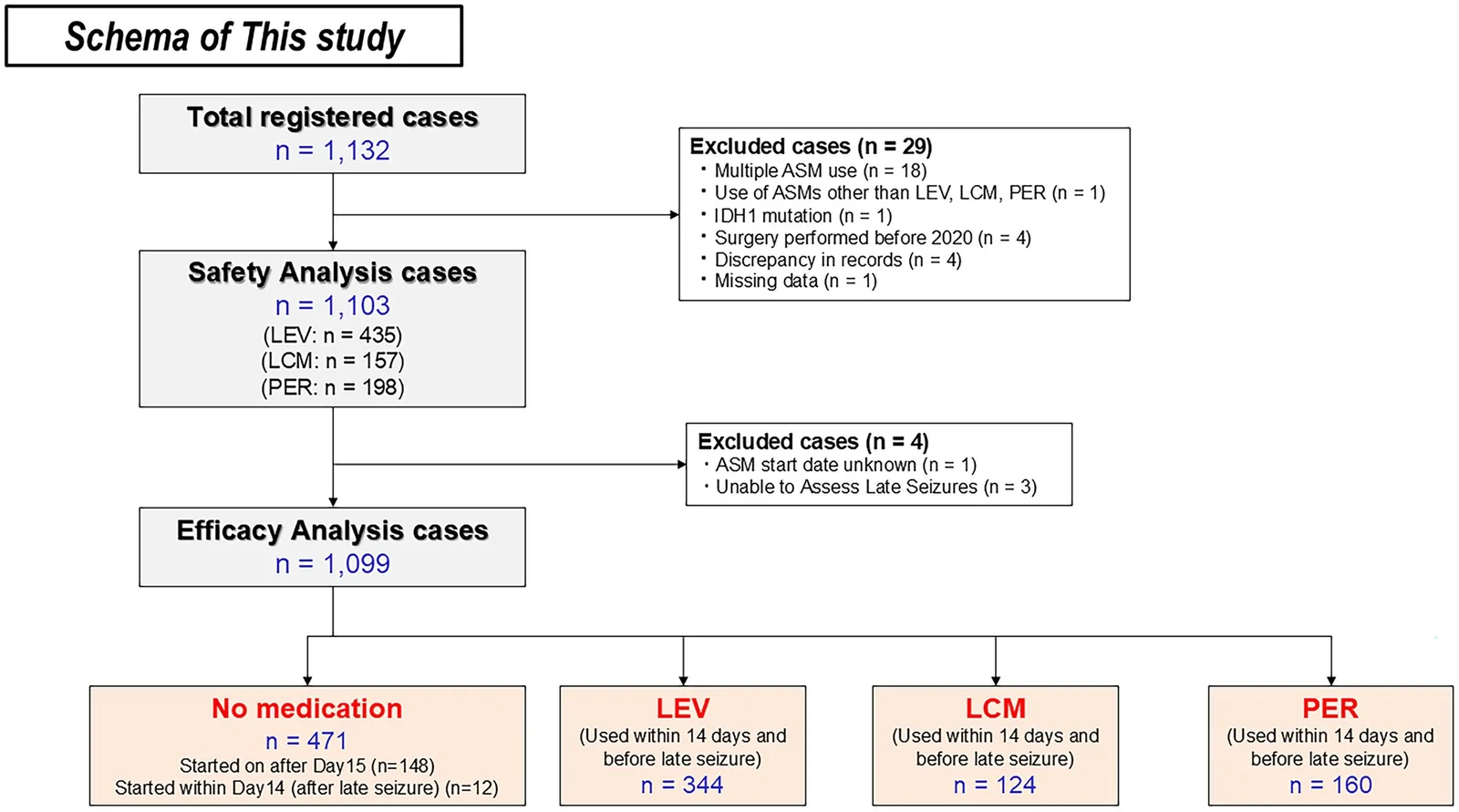
Flowchart of case selection and classification for safety and efficacy analyses. A total of 1132 cases were registered. After excluding 29 cases, 1103 were included in the safety analysis. Of these cases, 4 were excluded, resulting in 1099 cases for the efficacy analysis. Cases were categorized into 4 groups: no medication (*n* = 471), levetiracetam (LEV; *n* = 344), lacosamide (LCM; *n* = 124), and perampanel (PER; *n* = 160).

This study evaluated patient characteristics, cumulative incidence of late seizures, treatment regimens, including postoperative ASM use, and clinical outcomes such as seizure incidence, OS, and PFS. Early seizures were defined as those occurring within 7 days postoperatively, whereas late seizures were defined as those occurring 8 days or more after surgery. Informed consent for study participation was waived by each institutional review board owing to the retrospective, opt‑out design. The study schema is shown in Figure 1.

### Statistical Analysis

All time-to-event analyses (late seizure incidence, PFS, and OS) used postoperative day 8 as the time origin. PFS was defined as time to disease progression or death, and OS as time to death from any cause.

In the primary analysis, outcomes were analyzed according to the prespecified initial postoperative treatment strategy, regardless of subsequent treatment discontinuation, switching, the addition of concomitant ASMs, or ASM initiation in the no medication group; these treatment modifications were not treated as censoring events.

For late seizure incidence, cumulative incidence functions (CIFs) were estimated, treating death as a competing risk. CIFs were estimated nonparametrically using the Aalen–Johansen estimator. Two complementary approaches were used: (1) Fine–Gray models^12^^,^^13^ adjusting for tumor locations, preoperative seizure, and age to estimate sub-distribution hazard ratios (SHRs), and (2) overlap weighting based on generalized propensity scores to estimate CIFs at 6, 12, 18, and 24 months (bootstrap 95% confidence intervals [CIs], 2000 resamples) and weighted SHRs.

Generalized propensity scores for the 4 ASM groups were estimated using multinomial logistic regression including age, sex, KPS score, preoperative seizure, tumor location, resection rate, O-6-methylguanine-DNAmethyltransferase, and carmustine wafer.^14^ Overlap weights were calculated as the product of all generalized propensity scores divided by the propensity score for the received treatment, targeting the overlap population with an optimal covariate balance.^15^ Balance was assessed using standardized mean differences, with values < 0.1 indicating an adequate balance (Supplementary Tables S1 and S2; Supplementary Figure S1).

Kaplan–Meier methods were used to estimate survival curves for PFS and OS. Hazard ratios (HRs) with 95% CIs were calculated using Cox proportional hazards models (multivariable-adjusted and overlap-weighted). Given the potential for treatment effects and covariate influences to differ across follow-up periods, exploratory piecewise Cox proportional hazards models were used. Time intervals (0-12, 12-24, and ≥ 24 months) were defined to capture early, intermediate, and late effects, with interval-specific HRs estimated for each period.

Additional analyses were performed to aid interpretation of the primary analysis. A supplementary while-on-initial-strategy analysis was performed only for late seizure ­incidence, in which treatment discontinuation or ASM initiation in the no-medication group was treated as a censoring event at the time of occurrence. An exploratory analysis using a stricter definition of the no medication group, restricted to patients who had never received any ASM during follow-up, was also performed only for late seizure incidence.

Subgroup analyses restricted to patients without preoperative seizures and dose-based analyses were performed for late seizure incidence and OS. In these additional analyses, regression models were adjusted for the same baseline covariates used in the corresponding primary multivariable models.

*P*-values < .05 were considered to indicate statistical significance. All analyses were performed using Stata SE version 19 (StataCorp LLC, College Station, TX).

## Results

### Patient Characteristics and Clinical Features

Between January 2021 and December 2023, 1132 consecutive cases of primary GBM with surgical treatment followed by radio-chemotherapy at 39 centers were initially registered. After applying the eligibility criteria, 1103 cases were included in the safety analysis. Of these cases, 4 were excluded from the efficacy analysis because of insufficient outcome data, resulting in 1099 cases (482 females and 617 males) for the efficacy analysis. Patient characteristics and clinical features in this study are described in Table 1. The mean age at surgery was 67.2 years (range, 18-94 years). The tumor location was the frontal lobe in 430 cases (39.1%), temporal lobe in 361 (32.8%), parietal lobe in 217 (19.7%), occipital lobe in 54 (4.9%), insula in 28 (2.5%), and multiple locations in 9 (0.8%). All patients underwent craniotomy for tumor resection using an image-guided neuro-navigation system. The EOR was classified as GTR in 476 patients (43.3%), non-GTR in 399 (36.3%), and biopsy in 224 (20.4%). Before overlap weighting, substantial differences in baseline characteristics were observed across groups. Compared with the no medication group, the ASM groups included more patients with preoperative seizure (maximum standardized mean difference [SMD]: 0.727), younger age (maximum SMD: −0.254), higher KPS scores (maximum SMD: 0.335), and higher GTR and lower biopsy rates (maximum SMD: −0.506). After overlap weighting, baseline characteristics were well balanced across groups, with all SMDs < 0.1 (Supplementary Table S1, Supplementary Figure S1). The effective sample sizes after weighting were 295 (62.6% of original) for the no medication group, 289 (84.0%) for LEV, 114 (91.9%) for LCM, and 143 (89.4%) for PER (Supplementary Table S2).

**Table 1.** Characteristics of the patients enrolled in this study

	Antiseizure medications used after surgery				
	No medication	LEV	LCM	PER	Total
	*n* = 471	*n* = 344	*n* = 124	*n* = 160	*n* = 1099
Age (years)	68.7 (13.1)	65.4 (13.0)	67.3 (11.7)	66.8 (12.3)	67.2 (12.9)
≤65	152 (32.3%)	152 (44.2%)	51 (41.1%)	61 (38.1%)	416 (37.9%)
≥66	319 (67.7%)	192 (55.8%)	73 (58.9%)	99 (61.9%)	683 (62.1%)
Sex					
Male	249 (52.9%)	196 (57.0%)	76 (61.3%)	96 (60.0%)	617 (56.1%)
Female	222 (47.1%)	148 (43.0%)	48 (38.7%)	64 (40.0%)	482 (43.9%)
Tumor location					
Frontal lobe	189 (40.1%)	135 (39.2%)	43 (34.7%)	63 (39.4%)	430 (39.1%)
Temporal lobe	150 (31.8%)	120 (34.9%)	39 (31.5%)	52 (32.5%)	361 (32.8%)
Parietal lobe	89 (18.9%)	66 (19.2%)	29 (23.4%)	33 (20.6%)	217 (19.7%)
Others	43 (9.1%)	23 (6.7%)	13 (10.5%)	12 (7.5%)	91 (8.3%)
Preoperative seizure					
No	441 (93.6%)	228 (66.3%)	86 (69.4%)	129 (80.6%)	884 (80.4%)
Yes	30 (6.4%)	116 (33.7%)	38 (30.6%)	31 (19.4%)	215 (19.6%)
Preoperative ASM administration					
No medication	389 (82.6%)	105 (30.5%)	31 (25.0%)	46 (28.7%)	571 (52.0%)
LEV	53 (11.3%)	231 (67.2%)	11 (8.9%)	16 (10.0%)	311 (28.3%)
LCM	11 (2.3%)	3 (0.9%)	81 (65.3%)	2 (1.2%)	97 (8.8%)
PER	12 (2.5%)	5 (1.5%)	1 (0.8%)	95 (59.4%)	113 (10.3%)
Others	6 (1.3%)	0 (0.0%)	0 (0.0%)	0 (0.0%)	6 (0.5%)
LEV/LCM	0 (0.0%)	0 (0.0%)	0 (0.0%)	1 (0.6%)	1 (0.1%)
Early seizure					
No	463 (98.3%)	318 (92.4%)	110 (88.7%)	148 (92.5%)	1039 (94.5%)
Yes	8 (1.7%)	26 (7.6%)	14 (11.3%)	12 (7.5%)	60 (5.5%)
KPS					
<70	206 (43.7%)	120 (34.9%)	39 (31.5%)	44 (27.5%)	409 (37.2%)
70-90	168 (35.7%)	132 (38.4%)	52 (41.9%)	78 (48.8%)	430 (39.1%)
≥90	97 (20.6%)	92 (26.7%)	33 (26.6%)	38 (23.8%)	260 (23.7%)
Resection rate					
GTR	175 (37.2%)	149 (43.3%)	58 (46.8%)	94 (58.8%)	476 (43.3%)
Non-GTR	170 (36.1%)	133 (38.7%)	43 (34.7%)	53 (33.1%)	399 (36.3%)
Biopsy	126 (26.8%)	62 (18.0%)	23 (18.5%)	13 (8.1%)	224 (20.4%)
Carmustine wafer					
No	429 (91.1%)	298 (86.6%)	105 (84.7%)	125 (78.1%)	957 (87.1%)
Yes	42 (8.9%)	46 (13.4%)	19 (15.3%)	35 (21.9%)	142 (12.9%)
PDT					
No	464 (98.5%)	330 (95.9%)	119 (96.0%)	154 (96.2%)	1067 (97.1%)
Yes	7 (1.5%)	14 (4.1%)	5 (4.0%)	6 (3.8%)	32 (2.9%)
MGMT					
Methylation	146 (31.0%)	117 (34.0%)	47 (37.9%)	59 (36.9%)	369 (33.6%)
Unmethylation	198 (42.0%)	160 (46.5%)	47 (37.9%)	55 (34.4%)	460 (41.9%)
Unknown	127 (27.0%)	67 (19.5%)	30 (24.2%)	46 (28.7%)	270 (24.6%)
TERT promotor mutation					
No	89 (18.9%)	60 (17.4%)	21 (16.9%)	17 (10.6%)	187 (17.0%)
Yes	173 (36.7%)	140 (40.7%)	40 (32.3%)	60 (37.5%)	413 (37.6%)
Unknown	209 (44.4%)	144 (41.9%)	63 (50.8%)	83 (51.9%)	499 (45.4%)
Bevacizumab					
No	207 (43.9%)	183 (53.2%)	68 (54.8%)	80 (50.0%)	538 (49.0%)
Yes	264 (56.1%)	157 (45.6%)	56 (45.2%)	78 (48.8%)	555 (50.5%)
Unknown	0 (0.0%)	4 (1.2%)	0 (0.0%)	2 (1.2%)	6 (0.5%)
Novo-TTF					
No	391 (83.0%)	289 (84.0%)	110 (88.7%)	124 (77.5%)	914 (83.2%)
Yes	79 (16.8%)	51 (14.8%)	14 (11.3%)	35 (21.9%)	179 (16.3%)
Unknown	1 (0.2%)	4 (1.2%)	0 (0.0%)	1 (0.6%)	6 (0.5%)

Abbreviations: GTR, gross total resection; KPS, Karnofsky Performance Status; LCM, lacosamide; LEV, levetiracetam; MGMT, O-6-methylguanine-DNAmethyltransferase; Novo-TTF, novo-tumor treatment fields; PDT, photodynamic therapy; PER, perampanel; TERT, telomerase reverse transcriptase.

In this study, the maintenance doses of ASMs were as follows. For LEV, 1000 mg/day was administered to 298 patients (86.6%), while alternative doses were used in 46 (13.4%). For LCM, 200 mg/day was prescribed in 80 patients (64.5%), with other doses in 44 (35.5%). For PER, 2 mg/day was used in 93 patients (58.1%), 4 mg/day in 63 (39.4%), and other doses in 4 (2.5%) (Supplementary Table S3). Preoperative seizures were observed in 215 cases (19.6%), and preoperative ASM was administered in 528 (48.0%), consisting of LEV in 311 patients (28.3%), LCM in 97 (8.8%), PER in 113 (10.3%), and other agents in 6 (0.5%). Preoperative prophylactic ASM was administered in 325 cases (36.8%). The median follow-up time, estimated by the reverse Kaplan–Meier method, was 24.1 (95% CI: 22.8-25.5) months. In the follow-up periods, 60 patients developed early seizure and 287 presented late seizures. Postoperative ASM administration was performed in 628 cases (57.1%), with LEV used in 344 patients (31.3%), LCM in 124 (11.3%), and PER in 160 (14.6%). Among the total cohort of 1099 patients, 837 had neither preoperative seizure nor early postoperative seizure. Of these 837 individuals, postoperative prophylactic ASM was administered in 403 cases (48.1%). Overall, postoperative ASM was not administered postoperatively in 471 (42.9%) of the 1099 cases. Among the patients classified as the “no medication” group, 148 subsequently initiated ASM after postoperative day 15, and 12 started ASM within 14 days following the occurrence of late seizures. Of the 471 patients initially classified into the no medication group, 160 initiated ASM during follow-up, with a median time to ASM initiation of 3.2 months. For patients initially treated with ASM, the median duration of initial ASM use was 11.9 months in the LEV group, 14.6 months in the LCM group, and 15.8 months in the PER group; the initial ASM was discontinued in 46 (13.4%), 11 (8.9%), and 20 (12.5%) of patients, respectively.

### Adverse Events Generated by ASM Administration

Regarding the clinical course following ASM administration, adverse events were observed in 27 cases (3.4%). The breakdown was as follows: psychiatric symptoms in 12 cases (LEV: 8; LCM: 2; PER: 2), drug rash in 9 (LEV: 4; LCM: 2; PER: 3), hepatic or renal dysfunction in 4 (LEV: 4), hematologic toxicity in one (PER: 1), and other in one (LCM: 1).

### Late Seizure Control

**Figure 2.**
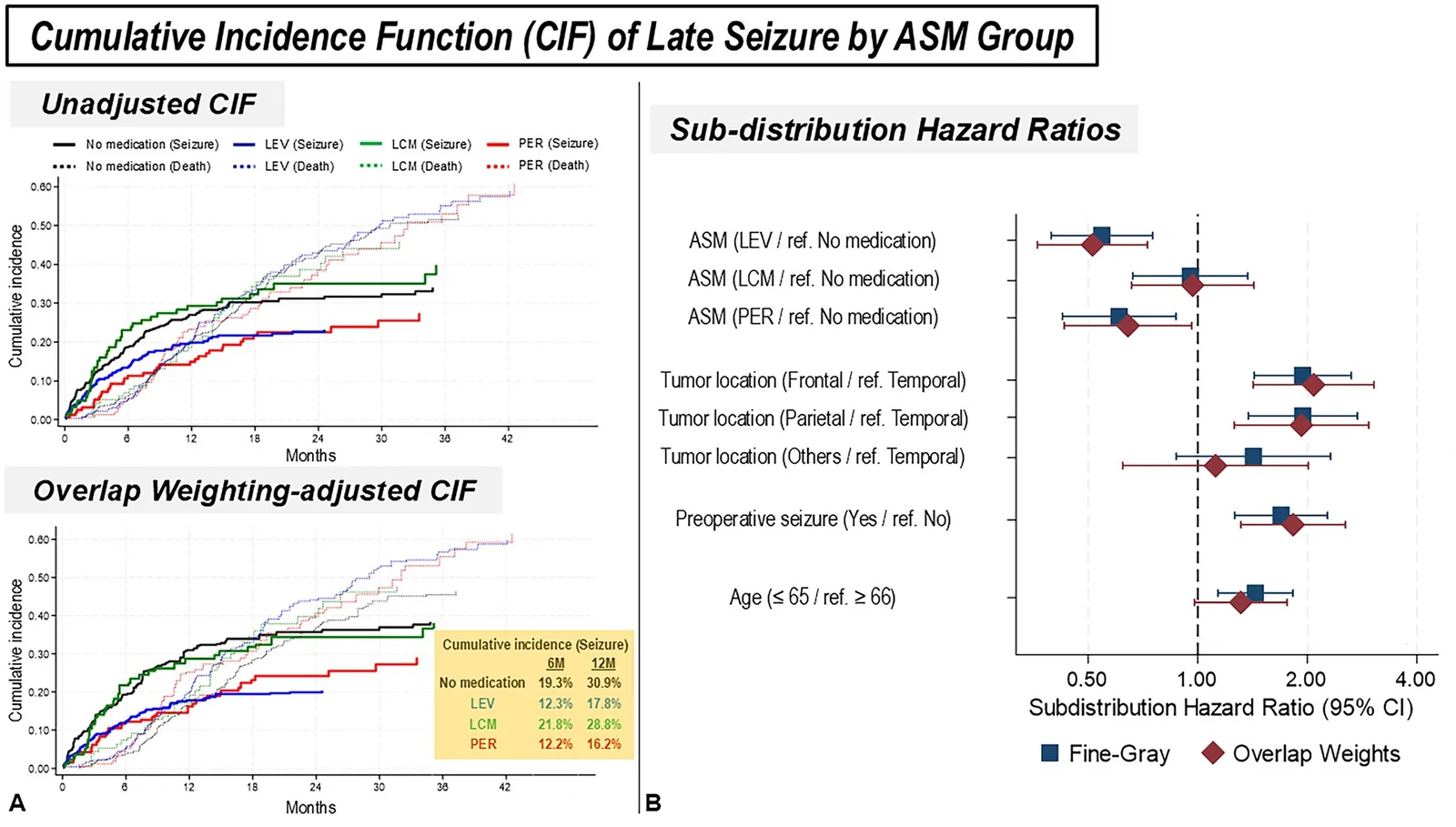
(A) Cumulative incidence of late seizures by ASM group, accounting for death as a competing risk. Upper panel: unadjusted cumulative incidence functions (CIFs). Lower panel: overlap-weighted CIFs. The 4 ASM groups are shown in different colors: no medication (black), LEV (blue), LCM (green), and PER (red). Within each ASM group, solid lines represent the cumulative incidence of late seizures (primary outcome) and dashed lines represent the cumulative incidence of death (competing risk event). (B) Sub-distribution hazard ratios (SHRs) and 95% confidence intervals for late seizures. Blue squares: Fine–Gray sub-distribution hazard models with covariate adjustment. Dark red rhombus: overlap weighting based on generalized propensity scores. High-risk factors for late seizure include: (1) tumor location in the frontal or parietal lobe; (2) history of preoperative seizure; and (3) age ≤ 65 years.

The maintenance doses of each ASM are summarized in Supplementary Table S3. Postoperative seizures occurred in 60 patients (5.5%) as early seizure and in 287 patients as late seizure, with a mean time to onset of late seizure of 193.6 days. The cumulative incidence of late seizure at 6 months estimated using overlap weighting was as follows: no medication, 19.3% (95% CI, 13.2-25.9); LEV, 12.3% (95% CI, 7.1-17.4); LCM, 21.8% (95% CI, 11.2-33.1); and PER, 12.2% (95% CI, 4.4-20.8). Similarly, at 12 months, the cumulative incidence was: no medication, 30.9% (95% CI, 22.6-39.6); LEV, 17.8% (95% CI, 11.6-24.0); LCM, 28.8% (95% CI, 16.9-41.2); and PER, 16.2% (95% CI, 6.6-25.9) (Table 2). These findings suggest that LEV and PER tend to be more effective than LCM or no medication (Figure 2A). By contrast, among patients who received prophylactic ASMs despite having no preoperative or early postoperative seizures, the cumulative incidence at 6 months was 18.3% (95% CI, 12.5-24.4) in the no medication group, 10.9% (95% CI, 5.3-17.4) in the LEV group, 16.3% (95% CI, 4.8-29.4) in the LCM group, and 8.3% (95% CI, 2.0-16.4) in the PER group. At 12 months, the cumulative incidence was 25.8% (95% CI, 18.8-32.4) for no medication, 16.8% (95% CI, 9.5-24.5) for LEV, 21.6% (95% CI, 7.4-36.4) for LCM, and 10.6% (95% CI, 2.9-19.4) for PER.

**Table 2.** Cumulative incidence (95% CI) at prespecified time points estimated using overlap weighting

	**No medication**		**LEV**		**LCM**		**PER**	
Months (M)	CIF	95% CI	CIF	95% CI	CIF	95% CI	CIF	95% CI
6	0.193	(0.132-0.259)	0.123	(0.071-0.174)	0.218	(0.112-0.331)	0.122	(0.044-0.208)
12	0.309	(0.226-0.396)	0.178	(0.116-0.240)	0.288	(0.169-0.412)	0.162	(0.066-0.259)
18	0.339	(0.258-0.426)	0.195	(0.130-0.257)	0.318	(0.199-0.442)	0.231	(0.124-0.339)
24	0.357	(0.273-0.447)	0.199	(0.135-0.262)	0.344	(0.216-0.478)	0.242	(0.130-0.352)

Abbreviations: CI, confidence interval; CIF, cumulative incidence function; LCM, lacosamide; LEV, levetiracetam; M, month; PER, perampanel.

In multivariable Fine–Gray models adjusted for age, tumor location, and preoperative seizure, LEV and PER were significantly associated with a lower cumulative incidence of late seizure compared with no medication (adjusted SHR for LEV: 0.55 [95% CI, 0.40-0.75], *P* < .001; for PER: 0.61 [95% CI, 0.42-0.87], *P* < .007), whereas LCM showed no significant difference (SHR: 0.95 [95% CI, 0.66-1.37], *P* = .798). In the overlap-weighted analysis, similar findings were observed: LEV (SHR: 0.51 [95% CI, 0.36-0.73], *P* < .001) and PER (SHR: 0.64 [95% CI, 0.43-0.96], *P* = .032) were associated with a lower cumulative incidence of late seizure compared with no medication, while LCM (SHR: 0.97 [95% CI, 0.66-1.43], *P* = .871) showed no difference. High-risk factors for late seizures included: 1) tumor location in the frontal or parietal lobe; 2) history of preoperative seizure; and 3) age ≤ 65 years (Figure 2B; Supplementary Table S4). We performed additional analyses to aid interpretation of the primary findings, including a supplementary while-on-initial-strategy analysis for late seizure incidence, an exploratory analysis restricted to patients who remained truly untreated, a subgroup analysis restricted to seizure-naive patients, and dose-based analyses of PER use stratified by dose (2 mg vs. ≥ 4 mg). Overall, these analyses yielded results broadly consistent with the primary findings (Supplementary Table S5).

### Outcomes (Antitumor Effect) by ASM Administration

**Figure 3.**
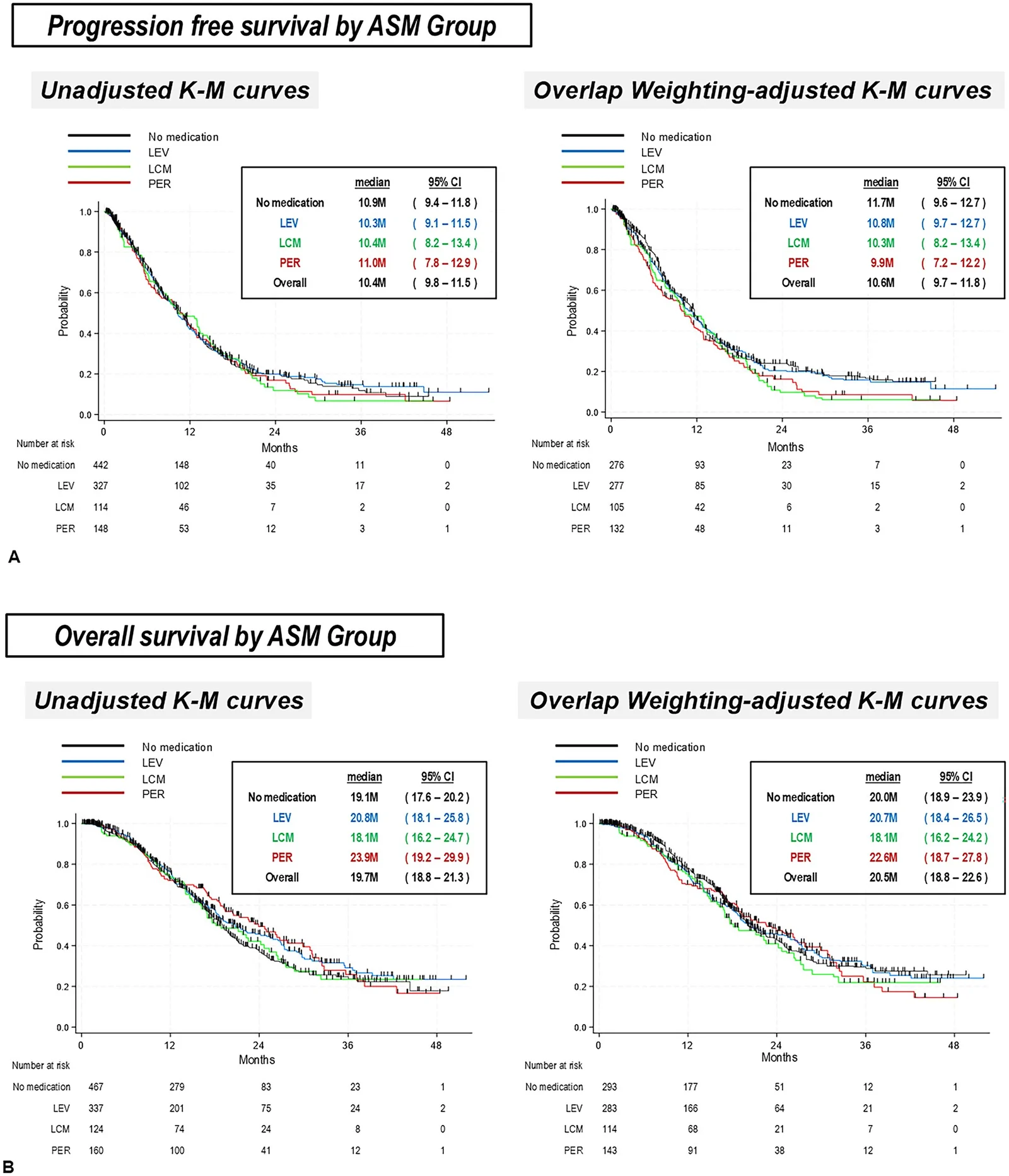
(A) Progression-free survival (PFS) and (B) overall survival (OS) according to postoperative ASM administration. Kaplan–Meier curves illustrate PFS and OS in patients with newly diagnosed GBM by postoperative ASM: no medication, LEV, LCM, and PER. Median PFS and OS with 95% confidence intervals (CIs) are shown for each group. For overlap-weighted analyses, 95% CIs were estimated using bootstrap resampling with 2000 resamples. Number at risk tables display actual patient counts for the unadjusted Kaplan–Meier curves and effective sample size (ESS) for the overlap-weighted curves. ESS represents the effective number of observations contributing to the weighted estimate.

Among the 1099 patients included in the analysis, the median PFS was 10.4 months (95% CI, 9.8-11.5) and the median OS was 19.7 months (95% CI, 18.8-21.3) (Figure 3A and B). In the multivariable Cox proportional hazards models, neither LEV (HR, 1.03 [95% CI, 0.87-1.23], *P* = .717) nor LCM (HR, 1.23 [95% CI, 0.96-1.56], *P* = .098) showed a significant difference in PFS compared with no medication, whereas PER was associated with shorter PFS (HR, 1.27 [95% CI, 1.02-1.59], *P* = .036). In the overlap-weighted analysis, similar associations were observed: LEV (HR, 1.02 [95% CI, 0.82-1.26], *P* = .866), LCM (HR, 1.26 [95% CI, 0.97-1.62], *P* = .078), and PER (HR, 1.26 [95% CI, 0.98-1.61], *P* = .070), all compared with no medication (Supplementary Table S6). For OS, multivariable Cox proportional hazards models showed no significant differences between any ASM group and no medication (HR for LEV: 0.93 [95% CI, 0.76-1.14], *P* = .485; LCM: 1.17 [95% CI, 0.89-1.54], *P* = .250; PER: 1.05 [95% CI, 0.81-1.35], *P* = .722). Similarly, in the overlap-weighted analysis, no significant differences in OS were observed (HR for LEV: 0.95 [95% CI, 0.75-1.21, *P* = .686]; LCM: 1.23 [95% CI, 0.92-1.64], *P* = .161; PER: 1.05 [95% CI, 0.79-1.38], *P* = .752) (Supplementary Table S7). Additional analyses for OS, including restriction to seizure-naive patients and dose stratification of PER (2 mg vs. ≥ 4 mg), yielded results broadly consistent with the primary analysis. No clear evidence of differences in OS across groups or a dose-dependent association with PER dose was observed (Supplementary Table S5).

### Time-Varying Effects of Each ASM on OS

In exploratory piecewise Cox proportional hazards models, the association between PER and OS varied substantially over time. Compared with no medication, PER showed opposing associations across follow-up intervals: a trend toward higher hazards during 0-12 months (HR, 1.41 [95% CI, 0.97-2.05], *P* = .070), lower hazards during 12-24 months (HR, 0.65 [95% CI, 0.42-1.01], *P* = .054), and no significant difference during ≥ 24 months (HR, 1.45 [95% CI, 0.77-2.74], *P* = .250). LEV and LCM showed no significant associations with OS in any interval. Overlap-weighted analysis confirmed consistent time-varying patterns for PER (0-12 months: HR, 1.43 [95% CI, 0.98-2.08], *P* = .061; 12-24 months: HR, 0.61 [95% CI, 0.39-0.95], *P* = .030).

**Figure 4.**
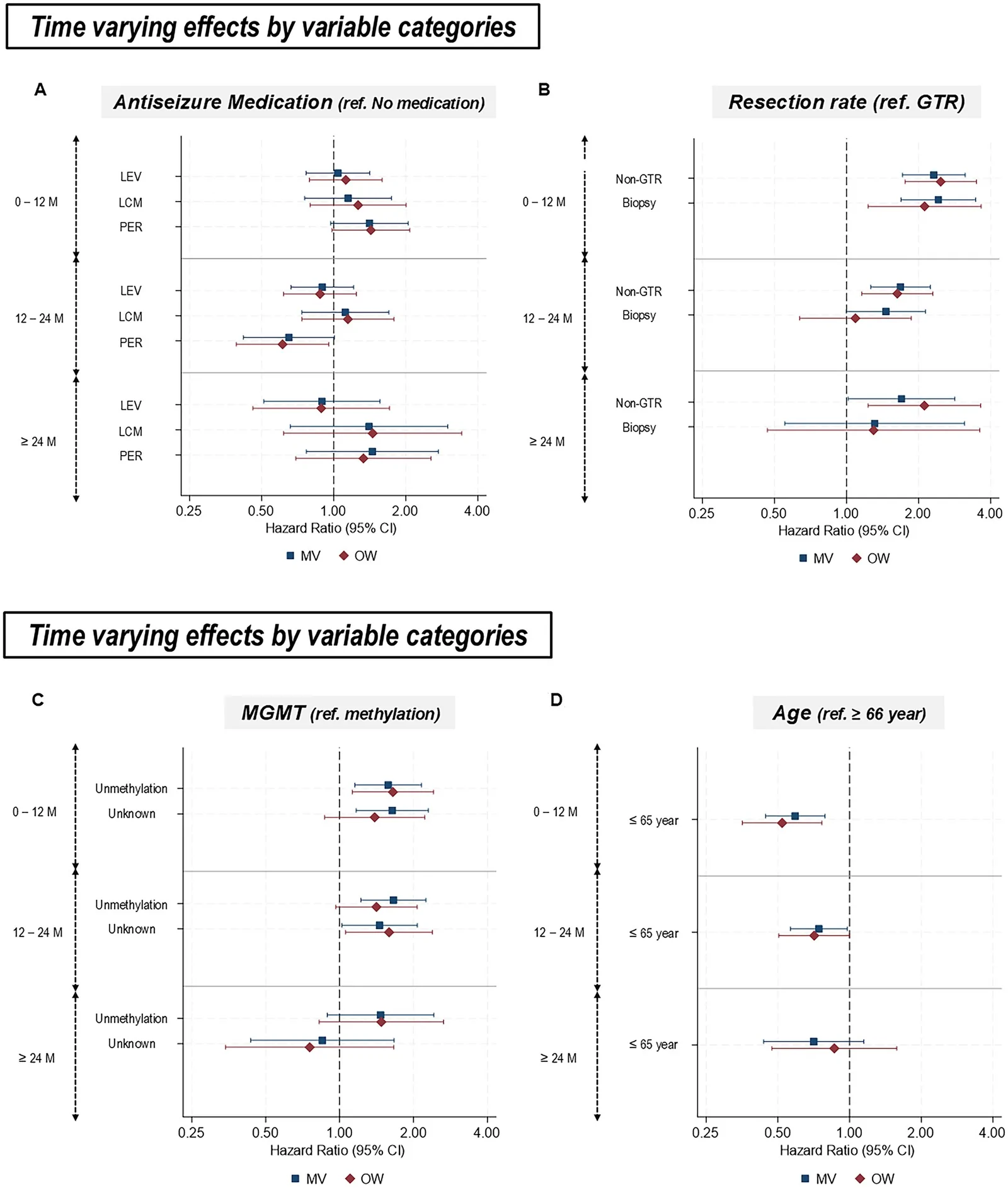

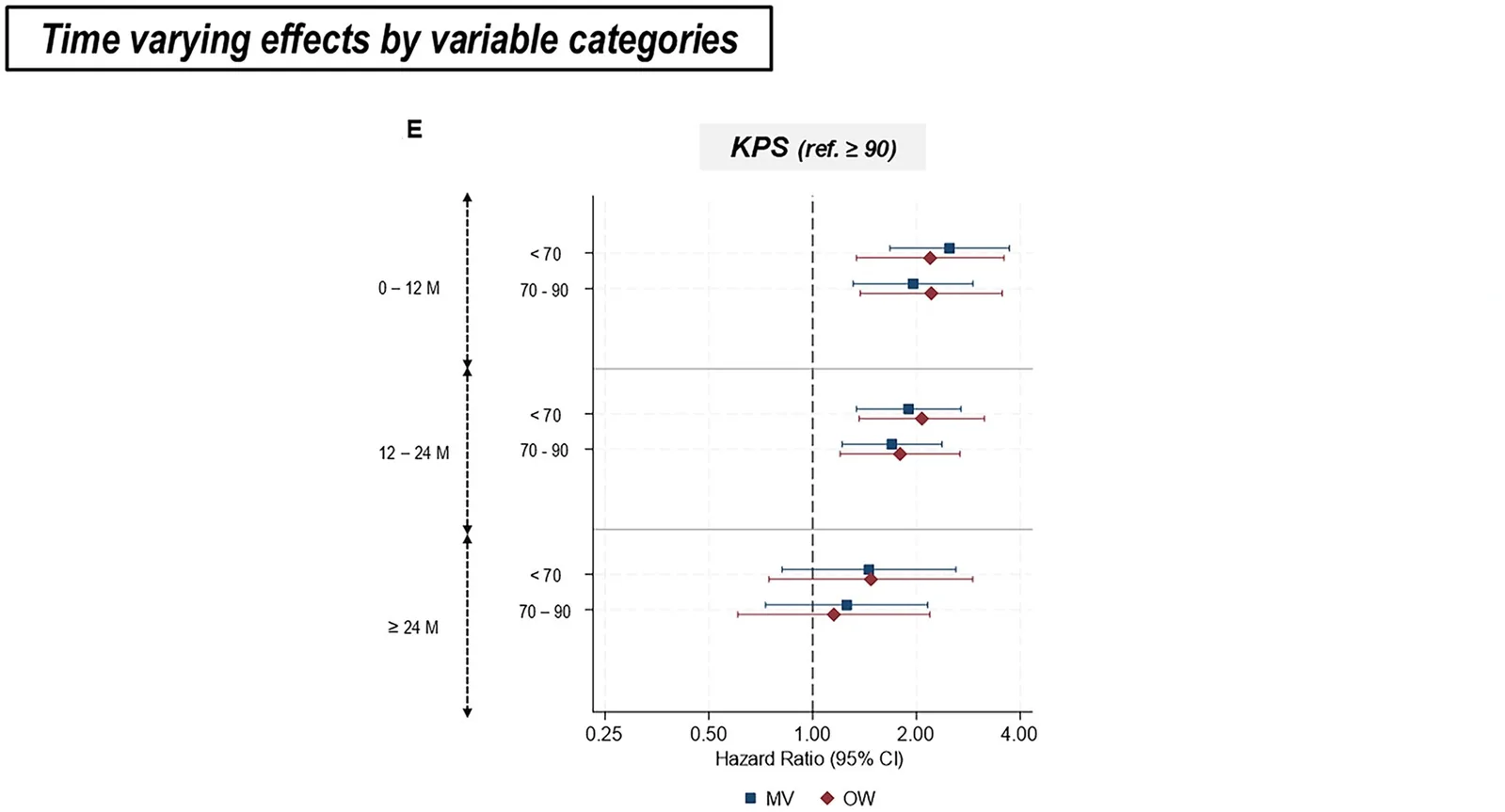
To assess time-varying changes in treatment efficacy, the observation period was divided into 3 intervals: (1) 0-12 months, (2) 12-24 months, and (3) ≥ 24 months. Within each interval, the effect of ASM, resection rate, O-6-methylguanine-DNAmethyltransferase (MGMT), age, and Karnofsky Performance Status (KPS) on OS were evaluated using piecewise Cox proportional hazards models with multivariable adjustment and overlap weighting. (A) ASM (ref. no medication), (B) Resection rate (ref. gross total resection [GTR]), (C) MGMT (ref. methylation), (D) Age (ref. ≥ 66 years), (E) KPS (ref. ≥ 90). Blue square: multivariable analysis. Dark-red rhombus: overlap-weighted analysis. M: months. ref. reference.

By contrast, the resection rate and KPS score showed strong associations predominantly during the 0-12 month interval. Compared with GTR, non-GTR (HR, 2.31 [95% CI, 1.71-3.13], *P* < .001) and biopsy (HR, 2.42 [95% CI, 1.69-3.46], *P* < .001) were associated with higher hazards during 0-12 months. Similarly, lower KPS scores were associated with higher hazards during this early period (KPS 70-90 vs. ≥ 90: HR, 1.96 [95% CI, 1.31-2.92], *P* = .001; KPS < 70 vs. ≥ 90: HR, 2.50 [95% CI, 1.67-3.72], *P* < .001). Overlap-weighted analysis showed consistent results for resection rate (non-GTR vs. GTR: HR, 2.47 [95% CI, 1.76-3.49], *P* < .001; biopsy vs. GTR: HR, 2.12 [95% CI, 1.23-3.65], *P* = .007) and KPS (KPS 70-90 vs. ≥ 90: HR, 2.21 [95% CI, 1.38-3.55], *P* = .001; KPS < 70 vs. ≥ 90: HR, 2.19 [95% CI, 1.34-3.59], *P* = .002) (Figure 4, Supplementary Table S8).

## Discussion

In this study, we investigated the real-world patterns of ASM use in Japan. The results indicated that LEV was the most frequently prescribed, followed by LCM and PER at comparable levels. Regarding late seizure control, LEV and PER demonstrated comparable effectiveness and showed a trend toward superior seizure control relative to no medication and LCM. In addition, neither the presence nor type of ASM was clearly associated with significant differences in PFS or OS, suggesting that ASM selection does not influence survival in patients with GBM.

Preserving QOL in the setting of limited life expectancy has become a critical matter in the treatment for patients with GBM. Effective late seizure control is integral to safeguarding QOL and is considered a priority alongside survival. Current treatment guidelines do not recommend a single established agent for BTRE. A systematic review and other studies have suggested LEV or lamotrigine monotherapy as first-line options for patients with primary or metastatic brain tumors presenting with seizure.^16^^,^^17^ On the other hand, Vacher et al.^17^ proposed carbamazepine, LCM, oxcarbazepine, PER, valproic acid, topiramate, and zonisamide as second-line alternatives. If adjunctive ASM therapy can meaningfully reduce the seizure burden and favorably influence survival, it could ultimately be incorporated into the standard of care for GBM. BTRE is a major complication of GBM and may occur throughout the disease course. Postoperative late seizure differs from preoperative seizure both mechanistically and prognostically. Although multiple studies have examined seizure frequency and management in patients with GBM, the results remain inconsistent.^18^^,^^19^ Prospective data on ASMs are limited, but many reports indicate efficacy for symptomatic seizure control.^19^^,^^20^ The role of prophylactic ASM administration in seizure‑free patients is unclear, despite the observation that approximately 10%-30% of such patients develop seizure during the course of illness.^2^ Importantly, evidence does not support the prophylactic use of ASMs in seizure‑free patients, and unnecessary drug exposure may adversely affect daily and social functioning.^21^ In this context, it is important to note that the American Academy of Neurology practice parameter advising against prophylactic ASM use in seizure-naive patients with brain tumor was established prior to the widespread availability of newer agents. Even with contemporary ASMs such as LEV and PER, our findings suggest that prophylactic administration may reduce, but not eliminate, the risk of late seizures. Moreover, given the pooled retrospective design of this study, we were unable to distinguish prophylactic from reactive ASM use clearly during follow-up. Therefore, our results should not be interpreted as justification to revise current guideline recommendations; rather, they suggest that if prophylactic ASM is used in clinical practice despite existing guidance, agents such as LEV or PER may be preferable options. Prospective studies incorporating QOL as a co-primary end point are warranted to define the role of prophylactic ASM more accurately in this setting. Conversely, surveys indicate that ASMs are frequently prescribed perioperatively to prevent complications and sustain treatment continuity and QOL.^21^^,^^22^ Notably, international real‑world practices show a pattern broadly similar to the present findings, with LEV widely regarded as the preferred first‑line ASM for BTRE because of its favorable interaction profile and tolerability, whereas second‑line choices such as LCM and PER vary across regions depending on regulatory approval, clinician familiarity, and institutional preference.^23^ In our multicenter retrospective cohort, postoperative ASMs were administered in 57.1% of cases, with prophylactic use in 48.1% of newly diagnosed GBM cases in Japan. LEV was the most frequently prescribed agent (31.3%), followed by PER (14.6%) and LCM (11.3%). At 12 months, cumulative late‑seizure incidence was 30.9% with no medication, 17.8% with LEV, 28.8% with LCM, and 16.2% with PER. Multivariable analysis revealed that LEV and PER displayed significantly lower cumulative incidences of late seizure than no medication and LCM. Taken together, LEV or PER may be preferred as postoperative ASMs to optimize seizure control and help preserve patients’ QOL. By contrast, prophylactic administration of ASM in patients with no history of any seizure attack reduced the rate of seizure occurrence, but it did not completely inhibit the occurrence of postoperative late seizure with any ASM. This finding indicates that the development of postoperative seizure may be caused by much more complicated mechanisms, including neuroexcitatory pathways.

In this study, we focused on 3 representative second-generation ASMs commonly used in Japan: LEV, LCM, and PER. LEV acts primarily via synaptic vesicle protein 2A, modulating neurotransmitter release and dampening neuronal hyperexcitability.^24^ The minimal drug–drug interactions of LEV favor use alongside chemotherapeutic agents,^25^ and its tolerability with relatively low cognitive impact supports QOL preservation.^26^ Behavioral adverse effects (eg irritability, mood changes, rarely psychosis) can complicate management, and evidence for its superiority in preventing late‑onset seizure after GBM surgery remains inconclusive.^26^ LCM enhances the slow inactivation of voltage‑gated sodium channels, stabilizing hyperexcitable membranes.^27^ Its favorable pharmacokinetics, limited interactions, and intravenous and oral formulations make it suitable in the perioperative setting. Clinical data support efficacy and tolerability in tumor‑related epilepsy, particularly as add‑on therapy when first‑line agents such as LEV are insufficient. However, large prospective trials are scarce, and its role in postoperative seizure prevention in GBM requires further study.^26^ On the other hand, PER selectively antagonizes AMPA receptors, inhibiting glutamate‑mediated activation and thereby reducing neuronal hyperexcitability and focal as well as tonic–clonic seizures.^28^ Malignant gliomas display altered glutamatergic signaling: upregulation of the cystine/glutamate transporter (xCT) increases extracellular glutamate release, while reduced EAAT2 expression impairs uptake.^29^ Excess extracellular glutamate in GBM not only promotes seizures via AMPA receptor activation, but may also drive proliferation and invasion through calcium‑permeable AMPA channels.^28^^,^^29^ In our prior work, magnetic resonance spectroscopy‑based quantification showed that elevated glutamate at the tumor margin was associated with postoperative BTRE.^4^ Preclinical studies further suggest antitumor effects of PER by interrupting neuron‑to‑glioma signaling,^30^ although its antitumor efficacy in clinical practice remains unclear. Clinical trials in focal epilepsy have demonstrated robust seizure freedom rates with PER monotherapy in adolescents and adults,^31^ underscoring its antiseizure potential.

In the present study, postoperative PER was associated with a reduced incidence of late seizure (12.2% at 6 months and 16.2% at 12 months), suggesting its potential clinical utility for seizure prevention. Although PER did not improve PFS or OS in the overall analysis, an apparent OS advantage was observed during the 12-24 months interval. This finding may raise the possibility that seizure control may indirectly influence survival or that certain subgroups could derive greater benefit. Thus, 2 key questions emerge: why did LCM show lower efficacy than PER and LEV, and why did OS appear to be longer in the PER group during months 12-24? First, differences in mechanisms of action may be relevant. LEV and PER primarily modulate synaptic neurotransmission, including glutamatergic pathways,^24^^,^^28^ whereas LCM acts by enhancing the slow inactivation of voltage-gated sodium channels.^27^ If glutamate-driven epileptogenesis plays a role in GBM, LEV and PER may be more likely to suppress these processes. Second, our previous work linked GBM recurrence to increased extracellular glutamate via xCT upregulation, which has been suggested to contribute to late seizures.^29^ In the present study, median PFS was approximately 10-11 months, and the mean time to late seizure onset was about 194 days, which may be consistent with the notion that postoperative late seizures can serve as a potential clinical indicator of GBM recurrence. Although the relative superiority of LEV vs. PER cannot be determined, our time-dependent analysis suggested a possible trend toward greater OS benefit with PER. This observation may be consistent with its glutamate-selective mechanism and supports further consideration of PER as an adjuvant ASM following surgery for GBM. Moreover, the NOA‑30/PerSurge phase IIa trial testing perioperative PER in recurrent GBM is expected to clarify whether AMPA antagonism can translate into clinically meaningful antitumor activity and survival benefit.^32^

However, several limitations should be considered when interpreting present findings. First, as this was an observational study, causal inference is limited. Despite overlap weighting adjustment, residual confounding by unmeasured factors cannot be excluded and may explain observed associations. Second, potential informative censoring bias may have influenced our results. Approximately 12.7% of the patients were censored within 6 months likely reflecting disease progression, clinical deterioration, or withdrawal from active care rather than random administrative censoring. Such informative censoring can introduce substantial bias in survival estimates, particularly for PFS. Third, the “No medication” group was defined according to initial postoperative ASM status. As some patients initiated ASM later during follow-up, potential treatment contamination may have occurred. In addition, detailed information on medication adherence, dose modifications, treatment duration, and switches between ASMs was not systematically available. These treatment dynamics, which could influence comparative effectiveness, represent an important limitation. However, due to inconsistent data capture across institutions, we were unable to perform sensitivity analyses restricted to patients with strictly documented continuous monotherapy. Given the lack of detailed time-dependent treatment data, further restriction to strictly untreated patients was not feasible and may have introduced additional bias. These treatment pattern variables could potentially influence effectiveness, but were inadequately captured in our dataset, limiting comprehensive assessment of treatment effects. Fourth, the sample sizes for PER and LCM were smaller than that for LEV, potentially limiting the statistical power for detecting differences and reducing precision in subgroup and time-stratified analyses. In particular, the observed OS advantage for PER during months 12-24 was derived from an exploratory piecewise Cox analysis with borderline statistical significance and should therefore be interpreted cautiously as hypothesis-generating rather than confirmatory. Finally, our study was not designed to inform or revise current clinical guidelines, as it lacks prospective validation and did not incorporate patient-centered outcomes such as QOL as a co-primary end point. Given these limitations of observational research, our findings warrant cautious interpretation. Future prospective studies incorporating comprehensive clinical data and QOL measures are needed to provide more definitive evidence and inform clinical decision-making.

We investigated the current status and clinical significance of ASM administration in postoperative patients with newly diagnosed GBM in Japan. LEV and PER were associated with a reduced risk of postoperative late seizures compared with LCM or no medication. Therefore, LEV or PER may be considered as postoperative ASMs to optimize seizure control and potentially help preserve patients’ QOL.

## Supplementary Material

vdag205_Supplementary_Data

## Data Availability

The raw data for this research can be obtained from Ehime University Hospital.
