# Supplementary material for: Role of antiseizure medication in postoperative late seizure and survival among patients with newly diagnosed glioblastoma: A multicenter retrospective study
Source: Neurooncol Adv. 2026 Aug 7;8(1):vdag205. doi: 10.1093/noajnl/vdag205 (PMC13493057; doi:10.1093/noajnl/vdag205)
Supplement: Supplementary file 1 [file vdag205_supplementary_data.zip › Supplementary Table S8.docx]

|  | **Supplementary Table S8.** Hazard ratios by time period using piecewise cox proportional hazards model | | | | | | | | | | | | | | | | |  |  | | |  |  |  | |  |  | |  |  |
| --- | --- | --- | --- | --- | --- | --- | --- | --- | --- | --- | --- | --- | --- | --- | --- | --- | --- | --- | --- | --- | --- | --- | --- | --- | --- | --- | --- | --- | --- | --- |
|  |  | |  | |  | |  |  |  |  |  |  |  |  |  |  |  |  |  | | |  |  |  | |  |  | |  |  |
|  | **(a)** Multivariable Cox PH model* | | | | | |  |  |  |  |  |  |  |  |  |  |  |  |  | | |  |  |  | |  |  | |  |  |
|  |  |  | |  | | |  |  |  |  |  |  |  |  |  |  |  |  |  | | |  |  |  | |  |  | |  |  |
|  |  |  | | **0–12 months** | | | | | | | | | **12–24 months** | | | | | | | | | **≥ 24 months** | | | | | | | | |
|  |  |  | | HR | |  | | 95% CI | | |  | p value | HR |  | 95% CI | | |  | p value | | | HR |  | | 95% CI | | | |  | p value |
|  |  |  | |  | |  | |  |  |  |  |  |  |  |  |  |  |  |  | | |  |  | |  |  |  | |  |  |
|  | **ASM** | **No medication** | | [reference] | |  | |  |  |  |  |  |  |  |  |  |  |  |  | | |  |  | |  |  |  | |  |  |
|  |  | **LEV** | | 1.04 | |  | | (0.77 | – | 1.41) |  | 0.795 | 0.90 |  | (0.66 | – | 1.21) |  | 0.475 | | | 0.89 |  | | (0.51 | – | 1.56) | |  | 0.692 |
|  |  | **LCM** | | 1.15 | |  | | (0.76 | – | 1.74) |  | 0.514 | 1.12 |  | (0.74 | – | 1.70) |  | 0.601 | | | 1.41 |  | | (0.66 | – | 2.99) | |  | 0.378 |
|  |  | **PER** | | 1.41 | |  | | (0.97 | – | 2.05) |  | 0.070 | 0.65 |  | (0.42 | – | 1.01) |  | 0.054 | | | 1.45 |  | | (0.77 | – | 2.74) | |  | 0.250 |
|  | **Resection rate** | **GTR** | | [reference] | |  | |  |  |  |  |  |  |  |  |  |  |  |  | | |  |  | |  |  |  | |  |  |
|  |  | **Non-GTR** | | 2.31 | |  | | (1.71 | – | 3.13) |  | <0.001 | 1.68 |  | (1.26 | – | 2.24) |  | <0.001 | | | 1.70 |  | | (1.02 | – | 2.84) | |  | 0.044 |
|  |  | **Biopsy** | | 2.42 | |  | | (1.69 | – | 3.46) |  | <0.001 | 1.46 |  | (1.00 | – | 2.14) |  | 0.051 | | | 1.31 |  | | (0.55 | – | 3.11) | |  | 0.538 |
|  | **MGMT** | **Methylation** | | [reference] | |  | |  |  |  |  |  |  |  |  |  |  |  |  | | |  |  | |  |  |  | |  |  |
|  |  | **Unmethylation** | | 1.58 | |  | | (1.15 | – | 2.16) |  | 0.004 | 1.66 |  | (1.22 | – | 2.25) |  | 0.001 | | | 1.47 |  | | (0.89 | – | 2.42) | |  | 0.132 |
|  |  | **Unknown** | | 1.64 | |  | | (1.17 | – | 2.30) |  | 0.004 | 1.45 |  | (1.02 | – | 2.07) |  | 0.038 | | | 0.85 |  | | (0.43 | – | 1.67) | |  | 0.636 |
|  | **Age** | **≥ 66** | | [reference] | |  | |  |  |  |  |  |  |  |  |  |  |  |  | | |  |  | |  |  |  | |  |  |
|  |  | **≤ 65** | | 0.59 | |  | | (0.44 | – | 0.79) |  | <0.001 | 0.74 |  | (0.56 | – | 0.98) |  | 0.034 | | | 0.71 |  | | (0.44 | – | 1.15) | |  | 0.160 |
|  | **KPS** | **≥ 90** | | [reference] | |  | |  |  |  |  |  |  |  |  |  |  |  |  | | |  |  | |  |  |  | |  |  |
|  |  | **70–90** | | 1.96 | |  | | (1.31 | – | 2.92) |  | 0.001 | 1.70 |  | (1.22 | – | 2.37) |  | 0.002 | | | 1.26 |  | | (0.73 | – | 2.16) | |  | 0.410 |
|  |  | **< 70** | | 2.50 | |  | | (1.67 | – | 3.72) |  | <0.001 | 1.90 |  | (1.34 | – | 2.69) |  | <0.001 | | | 1.46 |  | | (0.82 | – | 2.60) | |  | 0.204 |
|  |  |  | |  | |  | |  |  |  |  |  |  |  |  |  |  |  |  | | |  |  | |  |  |  | |  |  |
|  |  |  | |  | |  | |  |  |  |  |  |  |  |  |  |  |  |  | | |  |  | |  |  |  | |  |  |
|  | *Adjusted for resection rate (GTR, non-GTR, biopsy), MGMT (methylation, unmethylation, unknown), age (≤ 65, ≥ 66), and KPS score (≥ 90, 70–90, < 70) | | | | | | | | | | | | | | | | | | | | | | | | | | | | | |
|  |  | |  | |  | |  |  |  |  |  |  |  |  |  |  |  |  |  | |  | |  |  | |  |  | |  |  |
|  | **(b)** Overlap weighting | | | |  | |  |  |  |  |  |  |  |  |  |  |  |  |  | |  | |  |  | |  |  | |  |  |
|  |  | |  | |  | |  |  |  |  |  |  |  |  |  |  |  |  |  | |  | |  |  | |  |  | |  |  |
|  |  | |  | | **0–12 months** | | | | | | | | **12–24 months** | | | | | | | | **≥ 24 months** | | | | | | | | | |
|  |  | |  | | HR | |  | 95% CI | | |  | p-value | HR |  | 95% CI | | |  | p-value | | HR | |  | 95% CI | | | |  | | p-value |
|  |  | |  | |  | |  |  |  |  |  |  |  |  |  |  |  |  |  | |  | |  |  | |  |  |  | |  |
|  | **ASM** | | **No medication** | | [reference] | |  |  |  |  |  |  |  |  |  |  |  |  |  | |  | |  |  | |  |  |  | |  |
|  |  |  | **LEV** | | 1.12 | |  | (0.79 | – | 1.59) |  | 0.513 | 0.88 |  | (0.62 | – | 1.24) |  | 0.463 | | 0.89 | |  | (0.46 | | – | 1.71) |  | | 0.721 |
|  |  |  | **LCM** | | 1.26 | |  | (0.80 | – | 2.01) |  | 0.319 | 1.15 |  | (0.74 | – | 1.78) |  | 0.546 | | 1.46 | |  | (0.62 | | – | 3.42) |  | | 0.390 |
|  |  |  | **PER** | | 1.43 | |  | (0.98 | – | 2.08) |  | 0.061 | 0.61 |  | (0.39 | – | 0.95) |  | 0.030 | | 1.33 | |  | (0.69 | | – | 2.55) |  | | 0.389 |
|  | **Resection rate** | | **GTR** | | [reference] | |  |  |  |  |  |  |  |  |  |  |  |  |  | |  | |  |  | |  |  |  | |  |
|  |  |  | **Non-GTR** | | 2.47 | |  | (1.76 | – | 3.49) |  | <0.001 | 1.63 |  | (1.16 | – | 2.29) |  | 0.005 | | 2.11 | |  | (1.23 | | – | 3.63) |  | | 0.007 |
|  |  |  | **Biopsy** | | 2.12 | |  | (1.23 | – | 3.65) |  | 0.007 | 1.09 |  | (0.64 | – | 1.87) |  | 0.754 | | 1.30 | |  | (0.47 | | – | 3.60) |  | | 0.617 |
|  | **MGMT** | | **Methylation** | | [reference] | |  |  |  |  |  |  |  |  |  |  |  |  |  | |  | |  |  | |  |  |  | |  |
|  |  |  | **Unmethylation** | | 1.65 | |  | (1.13 | – | 2.41) |  | 0.010 | 1.41 |  | (0.96 | – | 2.07) |  | 0.077 | | 1.48 | |  | (0.83 | | – | 2.65) |  | | 0.189 |
|  |  |  | **Unknown** | | 1.39 | |  | (0.87 | – | 2.22) |  | 0.171 | 1.59 |  | (1.06 | – | 2.39) |  | 0.026 | | 0.75 | |  | (0.34 | | – | 1.66) |  | | 0.485 |
|  | **Age** | | **≥ 66** | | [reference] | |  |  |  |  |  |  |  |  |  |  |  |  |  | |  | |  |  | |  |  |  | |  |
|  |  |  | **≤ 65** | | 0.52 | |  | (0.35 | – | 0.77) |  | 0.001 | 0.71 |  | (0.50 | – | 1.00) |  | 0.049 | | 0.86 | |  | (0.47 | | – | 1.58) |  | | 0.633 |
|  | **KPS** | | **≥ 90** | | [reference] | |  |  |  |  |  |  |  |  |  |  |  |  |  | |  | |  |  | |  |  |  | |  |
|  |  |  | **70–90** | | 2.21 | |  | (1.38 | – | 3.55) |  | 0.001 | 1.79 |  | (1.20 | – | 2.67) |  | 0.004 | | 1.15 | |  | (0.61 | | – | 2.19) |  | | 0.665 |
|  |  |  | **< 70** | | 2.19 | |  | (1.34 | – | 3.59) |  | 0.002 | 2.08 |  | (1.37 | – | 3.15) |  | 0.001 | | 1.48 | |  | 0.75 | | – | 2.92) |  | | 0.262 |
|  |  | |  | |  | |  |  |  |  |  |  |  |  |  |  |  |  |  | |  | |  |  | |  |  |  | |  |
|  |  | |  | |  | |  |  |  |  |  |  |  |  |  |  |  |  |  | |  | |  |  | |  |  |  | |  |
|  | *Adjusted for resection rate (GTR, non-GTR, biopsy), MGMT (methylation, unmethylation, unknown), age (≤ 65, ≥ 66), and KPS score (≥ 90, 70–90, < 70) | | | | | | | | | | | | | | | | | | | | | | | | | | | | | |
|  | HR, hazard ratio | | | |  | |  |  |  |  |  |  |  |  |  |  |  |  |  |  | | |  |  | |  |  | |  |  |
|  | CI, confidence interval | | | |  | |  |  |  |  |  |  |  |  |  |  |  |  |  |  | | |  |  | |  |  | |  |  |
|  | ASM, antiseizure medication | | | |  | |  |  |  |  |  |  |  |  |  |  |  |  |  |  | | |  |  | |  |  | |  |  |
|  | PER, perampanel | | | |  | |  |  |  |  |  |  |  |  |  |  |  |  |  |  | | |  |  | |  |  | |  |  |
|  | LEV, levetiracetam | | | |  | |  |  |  |  |  |  |  |  |  |  |  |  |  |  | | |  |  | |  |  | |  |  |
|  | LCM, lacosamide | | | |  | |  |  |  |  |  |  |  |  |  |  |  |  |  |  | | |  |  | |  |  | |  |  |
|  | GTR, gross total resection | | | |  | |  |  |  |  |  |  |  |  |  |  |  |  |  |  | | |  |  | |  |  | |  |  |
|  | MGMT, O-6-methylguanine-DNAmethyltransferase | | | | | | | | | | | |  |  |  |  |  |  |  |  | | |  |  | |  |  | |  |  |
|  | KPS, Karnofsky Performance Status | | | | | |  |  |  |  |  |  |  |  |  |  |  |  |  |  | | |  |  | |  |  | |  |  |
