# Supplementary material for: Role of antiseizure medication in postoperative late seizure and survival among patients with newly diagnosed glioblastoma: A multicenter retrospective study
Source: Neurooncol Adv. 2026 Aug 7;8(1):vdag205. doi: 10.1093/noajnl/vdag205 (PMC13493057; doi:10.1093/noajnl/vdag205)
Supplement: Supplementary file 1 [file vdag205_supplementary_data.zip › Supplementary Table S1.docx]

| **Supplementary Table S1.** Covariate balance before and after overlap weighting | | | | | | |
| --- | --- | --- | --- | --- | --- | --- |
|  |  |  |  |  |  |  |
|  |  |  |  |  |  |  |
|  |  |  |  |  |  |  |
|  | **Standardized mean difference** | | | | | |
|  |  |  |  |  |  |  |
|  | **LEV** | | **LCM** | | **PER** | |
|  |  |  |  |  |  |  |
|  |  |  |  |  |  |  |
|  | **Before** | **After** | **Before** | **After** | **Before** | **After** |
|  |  |  |  |  |  |  |
| **Age** | -0.254 | 0.020 | -0.110 | 0.007 | -0.145 | 0.042 |
| **KPS** | 0.170 | 0.015 | 0.193 | 0.028 | 0.335 | 0.051 |
| **Sex** | -0.083 | 0.037 | -0.171 | 0.032 | -0.144 | 0.070 |
| **Preoperative seizure** | 0.727 | -0.036 | 0.658 | -0.049 | 0.396 | -0.035 |
| **Tumor location** |  |  |  |  |  |  |
| Temporal lobe | 0.064 | 0.030 | -0.009 | 0.046 | 0.014 | 0.039 |
| Parietal lobe | 0.007 | -0.012 | 0.110 | -0.031 | 0.043 | 0.011 |
| Others | -0.091 | -0.007 | 0.046 | 0.016 | -0.059 | 0.011 |
| **Resection rate** |  |  |  |  |  |  |
| Non-GTR | 0.053 | 0.029 | -0.030 | 0.004 | -0.062 | 0.033 |
| Biopsy | -0.211 | 0.025 | -0.197 | 0.021 | -0.506 | 0.052 |
| **MGMT** |  |  |  |  |  |  |
| Unmethyl | 0.090 | -0.051 | -0.084 | -0.012 | -0.158 | -0.043 |
| Unknown | -0.178 | 0.043 | -0.064 | -0.001 | 0.040 | 0.030 |
| **Carmustine wafer** | 0.142 | -0.031 | 0.197 | 0.012 | 0.365 | -0.006 |
|  |  |  |  |  |  |  |
|  |  |  |  |  |  |  |
| *Standardized mean difference < 0.10 indicates good balance | | | | |  |  |
| LEV, levetiracetam |  |  |  |  |  |  |
| LCM, lacosamide |  |  |  |  |  |  |
| KPS, Karnofsky Performance Status | | |  |  |  |  |
| GTR, gross total resection | |  |  |  |  |  |
| MGMT, O-6-methylguanine-DNAmethyltransferase | | | | |  |  |
