# Supplementary material for: Role of antiseizure medication in postoperative late seizure and survival among patients with newly diagnosed glioblastoma: A multicenter retrospective study
Source: Neurooncol Adv. 2026 Aug 7;8(1):vdag205. doi: 10.1093/noajnl/vdag205 (PMC13493057; doi:10.1093/noajnl/vdag205)
Supplement: Supplementary file 1 [file vdag205_supplementary_data.zip › Supplementary Table S2.docx]

| **Supplementary Table S2.** Characteristics after overlap weighting. | | |  |  |  |
| --- | --- | --- | --- | --- | --- |
|  |  |  |  |  |  |
|  |  |  |  |  |  |
|  | **Antiseizure medications used after surgery** | | | | |
|  |  |  |  |  |  |
|  | **No medication** | **LEV** | **LCM** | **PER** | **Max SMD** |
|  |  |  |  |  |  |
| **Effective sample size (ESS)** | 295 | 289 | 114 | 143 |  |
| **ESS/N ratio** | 0.626 | 0.840 | 0.919 | 0.894 |  |
|  |  |  |  |  |  |
| **Age, mean (SD), years** | 66.8 (13.2) | 67.1 (12.1) | 66.9 (11.6) | 67.4 (12.1) | 0.042 |
| **KPS, mean(SD)** | 70.7 (18.5) | 71.0 (18.0) | 71.2 (19.6) | 71.5 (15.7) | 0.051 |
| **Sex** |  |  |  |  | 0.070 |
| Male | 61.6% | 59.7% | 60.0% | 58.1% |  |
| Female | 38.4% | 40.3% | 40.0% | 41.9% |  |
| **Tumor location** |  |  |  |  | 0.046 |
| Frontal lobe | 38.3% | 37.6% | 37.0% | 35.8% |  |
| Temporal lobe | 32.4% | 33.8% | 34.6% | 34.2% |  |
| Parietal lobe | 21.1% | 20.6% | 19.9% | 21.6% |  |
| Others | 8.2% | 8.0% | 8.6% | 8.5% |  |
| **Preoperative seizure** |  |  |  |  | 0.049 |
| No | 78.3% | 79.8% | 80.3% | 79.8% |  |
| Yes | 21.7% | 20.2% | 19.7% | 20.2% |  |
| **Resection rate** |  |  |  |  | 0.052 |
| GTR | 51.5% | 49.2% | 50.6% | 48.1% |  |
| Non-GTR | 35.6% | 37.0% | 35.8% | 37.1% |  |
| Biopsy | 12.9% | 13.8% | 13.7% | 14.8% |  |
| **MGMT** |  |  |  |  | 0.051 |
| Methylation | 35.9% | 36.6% | 36.6% | 36.7% |  |
| Unmethylation | 41.1% | 38.6% | 40.5% | 39.0% |  |
| Unknown | 23.0% | 24.8% | 22.9% | 24.2% |  |
| **Carmustine wafer** |  |  |  |  | 0.043 |
| No | 83.7% | 84.8% | 83.3% | 83.9% |  |
| Yes | 16.3% | 15.2% | 16.7% | 16.1% |  |
|  |  |  |  |  |  |
|  |  |  |  |  |  |
| ESS, effective sample size |  |  |  |  |  |
| PER, perampanel |  |  |  |  |  |
| LEV, levetiracetam |  |  |  |  |  |
| LCM, lacosamide |  |  |  |  |  |
| SMD, standardized mean difference |  |  |  |  |  |
| SD, standard deviation |  |  |  |  |  |
| KPS, Karnofsky Performance Status | |  |  |  |  |
| GTR, gross total resection |  |  |  |  |  |
| MGMT, O-6-methylguanine-DNAmethyltransferase | | |  |  |  |
|  |  |  |  |  |  |
| Sample sizes reflect the effective sample size (ESS) after overlap weighting, calculated as (Σwᵢ)²/Σ(wᵢ²) within each group. Original sample sizes were 471 for No medication, 344 for LEV, 124 for LCM, and 160 for PER. ESS/N ratios indicate the proportion of statistical information retained after weighting. Max SMD represents the maximum standardized mean difference across all six pairwise comparisons among the four groups. An SMD < 0.1 indicates excellent covariate balance, and an SMD < 0.2 indicates acceptable balance. All covariates achieved excellent balance after weighting (max SMD < 0.1 for all variables). Detailed pairwise standardized mean differences for comparisons between No medication and each of the other three groups are presented in Supplemental Table S1, and visual assessment of covariate balance before and after weighting is shown in Supplemental Figure S1. | | | | | |
|  |  |  |  |  |  |
|  |  |  |  |  |  |
|  |  |  |  |  |  |
|  |  |  |  |  |  |
