# Supplementary material for: Role of antiseizure medication in postoperative late seizure and survival among patients with newly diagnosed glioblastoma: A multicenter retrospective study
Source: Neurooncol Adv. 2026 Aug 7;8(1):vdag205. doi: 10.1093/noajnl/vdag205 (PMC13493057; doi:10.1093/noajnl/vdag205)
Supplement: Supplementary file 1 [file vdag205_supplementary_data.zip › Supplementary Table S3.docx]

| **Supplementary Table S3.** Antiseizure medication (maintenance dose). | |
| --- | --- |
|  |  |
|  |  |
| **Antiseizure medication (maintenance dose)** | **Value (%)** |
|  |  |
|  |  |
| Levetiracetam | 344 |
| *1000 mg | 298 (86.6) |
| Others | 46 (13.4) |
| Lacosamide (200 mg / others) | 124 |
| *200 mg | 80 (64.5) |
| Others | 44 (35.5) |
| Perampanel (2 mg / 4 mg / others) | 160 |
| *4 mg | 63 (39.4) |
| 2 mg | 93 (58.1) |
| Others | 4 (2.5) |
|  |  |
|  |  |
| *Recommended dose |  |
| Values represent the number of patients and percentage unless otherwise indicated. | |
