# Supplementary material for: Role of antiseizure medication in postoperative late seizure and survival among patients with newly diagnosed glioblastoma: A multicenter retrospective study
Source: Neurooncol Adv. 2026 Aug 7;8(1):vdag205. doi: 10.1093/noajnl/vdag205 (PMC13493057; doi:10.1093/noajnl/vdag205)
Supplement: Supplementary file 1 [file vdag205_supplementary_data.zip › Supplementary Table S4.docx]

| **Supplementary Table S4.** Unadjusted analysis and multivariable-adjusted and overlap-weighted analyses for late seizure control. | | | | | | | | | | | | | | | | |  |  |  |  |  |  |
| --- | --- | --- | --- | --- | --- | --- | --- | --- | --- | --- | --- | --- | --- | --- | --- | --- | --- | --- | --- | --- | --- | --- |
|  |  |  |  |  |  |  |  |  |  |  |  |  |  |  |  |  |  |  |  |  |  |  |
|  |  |  |  |  |  |  |  |  |  |  |  |  |  |  |  |  |  |  |  |  |  |  |
|  |  | **Unadjusted** | | | | | | | **Multivariable Fine–Gray model*** | | | | | | | **Overlap weighting** | | | | | | |
|  |  | SHR |  | 95% CI | | |  | p-value | SHR |  | 95% CI | | |  | p-value | SHR |  | 95% CI | | |  | p-value |
|  |  |  |  |  |  |  |  |  |  |  |  |  |  |  |  |  |  |  |  |  |  |  |
| **ASM** | **No medication** | [reference] |  |  |  |  |  |  |  |  |  |  |  |  |  |  |  |  |  |  |  |  |
|  | **LEV** | 0.68 |  | (0.51 | – | 0.91) |  | 0.009 | 0.55 |  | (0.40 | – | 0.75) |  | <0.001 | 0.51 |  | (0.36 | – | 0.73) |  | <0.001 |
|  | **LCM** | 1.14 |  | (0.80 | – | 1.61) |  | 0.471 | 0.95 |  | (0.66 | – | 1.37) |  | 0.798 | 0.97 |  | (0.66 | – | 1.43) |  | 0.871 |
|  | **PER** | 0.68 |  | (0.47 | – | 0.98) |  | 0.037 | 0.61 |  | (0.42 | – | 0.87) |  | 0.007 | 0.64 |  | (0.43 | – | 0.96) |  | 0.032 |
| **Tumor location** | **Temporal lobe** | [reference] |  |  |  |  |  |  |  |  |  |  |  |  |  |  |  |  |  |  |  |  |
|  | **Frontal lobe** | 1.97 |  | 1.45 |  | 2.68 |  | <0.001 | 1.94 |  | 1.43 |  | 2.64 |  | <0.001 | 2.08 |  | (1.42 | – | 3.05) |  | <0.001 |
|  | **Parietal lobe** | 1.97 |  | 1.39 |  | 2.78 |  | <0.001 | 1.94 |  | 1.38 |  | 2.74 |  | <0.001 | 1.93 |  | (1.26 | – | 2.95) |  | 0.003 |
|  | **Others** | 1.49 |  | 0.91 |  | 2.43 |  | 0.112 | 1.42 |  | 0.87 |  | 2.32 |  | 0.158 | 1.12 |  | (0.62 | – | 2.01) |  | 0.708 |
| **Preoperative seizure** | **No** | [reference] |  |  |  |  |  |  |  |  |  |  |  |  |  |  |  |  |  |  |  |  |
|  | **Yes** | 1.52 |  | 1.17 |  | 1.98 |  | 0.002 | 1.69 |  | 1.26 |  | 2.27 |  | <0.001 | 1.83 |  | (1.31 | – | 2.54) |  | <0.001 |
| **Age** | **≥ 66** | [reference] |  |  |  |  |  |  |  |  |  |  |  |  |  |  |  |  |  |  |  |  |
|  | **≤ 65** | 1.38 |  | 1.09 |  | 1.75 |  | 0.007 | 1.44 |  | 1.14 |  | 1.83 |  | 0.003 | 1.31 |  | (0.98 | – | 1.76) |  | 0.068 |
|  |  |  |  |  |  |  |  |  |  |  |  |  |  |  |  |  |  |  |  |  |  |  |
|  |  |  |  |  |  |  |  |  |  |  |  |  |  |  |  |  |  |  |  |  |  |  |
| *Adjusted for age (≤ 65, ≥ 66), tumor location (frontal lobe, temporal lobe, parietal lobe, others), preoperative seizure (yes, no) | | | | | | | | | | | | | | | | | | |  |  |  |  |
| SHR: sub-distribution hazard ratio | |  |  |  |  |  |  |  |  |  |  |  |  |  |  |  |  |  |  |  |  |  |
| CI, confidence interval | |  |  |  |  |  |  |  |  |  |  |  |  |  |  |  |  |  |  |  |  |  |
| PER, perampanel |  |  |  |  |  |  |  |  |  |  |  |  |  |  |  |  |  |  |  |  |  |  |
| LEV, levetiracetam |  |  |  |  |  |  |  |  |  |  |  |  |  |  |  |  |  |  |  |  |  |  |
| LCM, lacosamide |  |  |  |  |  |  |  |  |  |  |  |  |  |  |  |  |  |  |  |  |  |  |
