# Supplementary material for: Role of antiseizure medication in postoperative late seizure and survival among patients with newly diagnosed glioblastoma: A multicenter retrospective study
Source: Neurooncol Adv. 2026 Aug 7;8(1):vdag205. doi: 10.1093/noajnl/vdag205 (PMC13493057; doi:10.1093/noajnl/vdag205)
Supplement: Supplementary file 1 [file vdag205_supplementary_data.zip › Supplementary Table S5.docx]

**Supplementary Table S5.** Additional analyses for late seizure incidence and overall survival.

**(a)** Additional analysis for late seizure incidence

| **Analysis** | **Population/definition** | **ASM** | **No. of patients** | **No. of late seizures** | **Adjusted SHR*** | **95% CI** | **p-value** |
| --- | --- | --- | --- | --- | --- | --- | --- |
| **While-on-initial-strategy analysis** | **Censored at ASM initiation 　　　　　in the no medication group, discontinuation of ASMs** | **No medication** | 471 | 131 | [Reference] | - | - |
|  |  | **LEV** | 344 | 61 | 0.49 | 0.34–0.69 | <0.001 |
|  |  | **LCM** | 124 | 38 | 0.90 | 0.62–1.32 | 0.593 |
|  |  | **PER** | 160 | 27 | 0.49 | 0.33–0.72 | <0.001 |
| **Stricter no medication definition** | **No medication group restricted to patients who never received ASM during follow-up** | **No medication** | 428 | 131 | [Reference] | - | - |
|  |  | **LEV** | 344 | 70 | 0.48 | 0.34–0.67 | <0.001 |
|  |  | **LCM** | 124 | 41 | 0.84 | 0.58–1.23 | 0.382 |
|  |  | **PER** | 160 | 35 | 0.55 | 0.38–0.79 | 0.001 |
| **Subgroup without preoperative/early seizures** | **Patients without preoperative/early seizures** | **No medication** | 434 | 121 | [Reference] | - | - |
|  |  | **LEV** | 208 | 35 | 0.61 | 0.41–0.90 | 0.012 |
|  |  | **LCM** | 76 | 18 | 0.82 | 0.50–1.34 | 0.430 |
|  |  | **PER** | 119 | 21 | 0.55 | 0.35–0.86 | 0.008 |
| **Dose-stratified analysis** | **ASM groups defined as LEV, LCM, PER 2 mg, PER ≥ 4 mg** | **No medication** | 471 | 141 | [Reference] | - | - |
|  |  | **LEV** | 344 | 70 | 0.55 | 0.40–0.75 | <0.001 |
|  |  | **LCM** | 124 | 41 | 0.95 | 0.66–1.37 | 0.802 |
|  |  | **PER 2 mg** | 93 | 17 | 0.52 | 0.32–0.84 | 0.007 |
|  |  | **PER ≥ 4 mg** | 65 | 18 | 0.75 | 0.47–1.21 | 0.241 |

*Adjusted for age (≤ 65, ≥ 66 years), tumor location (frontal lobe, temporal lobe, parietal lobe, others), preoperative seizure (yes, no)

**(b)** Additional analysis for overall survival

| **Analysis** | **Population/definition** | **ASM** | **No. of patients** | **No. of deaths** | **Adjusted HR*** | **95% CI** | **p-value** |
| --- | --- | --- | --- | --- | --- | --- | --- |
| **Subgroup without preoperative/early seizures** | **Patients without preoperative/early seizures** | **No medication** | 434 | 230 | [Reference] | - | - |
|  |  | **LEV** | 208 | 94 | 0.90 | 0.70–1.15 | 0.392 |
|  |  | **LCM** | 76 | 43 | 1.23 | 0.89–1.71 | 0.215 |
|  |  | **PER** | 119 | 60 | 1.05 | 0.78–1.41 | 0.751 |
| **Dose-stratified analysis** | **ASM groups defined as LEV, LCM, PER 2 mg, PER ≥ 4 mg** | **No medication** | 471 | 249 | [Reference] | - | - |
|  |  | **LEV** | 344 | 165 | 0.93 | 0.76–1.14 | 0.487 |
|  |  | **LCM** | 124 | 66 | 1.18 | 0.89–1.55 | 0.247 |
|  |  | **PER 2 mg** | 93 | 43 | 0.90 | 0.65–1.25 | 0.535 |
|  |  | **PER ≥ 4 mg** | 65 | 39 | 1.27 | 0.90–1.80 | 0.171 |

*Adjusted for resection rate (GTR, non-GTR, biopsy), MGMT (methylation, unmethylation, unknown), age (≤ 65, ≥ 66 years), and KPS score (≥ 90, 70–90, < 70)
