# Supplementary material for: Role of antiseizure medication in postoperative late seizure and survival among patients with newly diagnosed glioblastoma: A multicenter retrospective study
Source: Neurooncol Adv. 2026 Aug 7;8(1):vdag205. doi: 10.1093/noajnl/vdag205 (PMC13493057; doi:10.1093/noajnl/vdag205)
Supplement: Supplementary file 1 [file vdag205_supplementary_data.zip › Supplementary Table S6.docx]

| **Supplementary Table S6.** Hazard ratios for progression-free survival by antiseizure medication. | | | | | | | | | | | | | | | |  |  |  |  |  |  |  |
| --- | --- | --- | --- | --- | --- | --- | --- | --- | --- | --- | --- | --- | --- | --- | --- | --- | --- | --- | --- | --- | --- | --- |
|  |  |  |  |  |  |  |  |  |  |  |  |  |  |  |  |  |  |  |  |  |  |  |
|  |  |  |  |  |  |  |  |  |  |  |  |  |  |  |  |  |  |  |  |  |  |  |
|  |  | **Unadjusted** | | | | | | | **Multivariable Cox PH model*** | | | | | | | **Overlap weighting** | | | | | | |
|  |  | HR |  | 95% CI | | |  | p value | HR |  | 95% CI | | |  | p value | HR |  | 95% CI | | |  | p value |
|  |  |  |  |  |  |  |  |  |  |  |  |  |  |  |  |  |  |  |  |  |  |  |
| **ASM** | **No medication** | [reference] |  |  |  |  |  |  |  |  |  |  |  |  |  |  |  |  |  |  |  |  |
|  | **LEV** | 0.97 |  | (0.82 | – | 1.16) |  | 0.735 | 1.03 |  | (0.87 | – | 1.23) |  | 0.717 | 1.02 |  | (0.82 | – | 1.26) |  | 0.866 |
|  | **LCM** | 1.05 |  | (0.82 | – | 1.33) |  | 0.714 | 1.23 |  | (0.96 | – | 1.56) |  | 0.098 | 1.26 |  | (0.97 | – | 1.62) |  | 0.078 |
|  | **PER** | 1.05 |  | (0.84 | – | 1.31) |  | 0.656 | 1.27 |  | (1.02 | – | 1.59) |  | 0.036 | 1.26 |  | (0.98 | – | 1.61) |  | 0.070 |
| **Resection rate** | **GTR** | [reference] |  |  |  |  |  |  |  |  |  |  |  |  |  |  |  |  |  |  |  |  |
|  | **Non-GTR** | 1.65 |  | (1.40 | – | 1.95) |  | <0.001 | 1.65 |  | (1.39 | – | 1.95) |  | <0.001 | 1.75 |  | (1.45 | – | 2.12) |  | <0.001 |
|  | **Biopsy** | 1.77 |  | (1.44 | – | 2.16) |  | <0.001 | 1.79 |  | (1.45 | – | 2.22) |  | <0.001 | 1.57 |  | (1.13 | – | 2.18) |  | 0.007 |
| **MGMT** | **Methylation** | [reference] |  |  |  |  |  |  |  |  |  |  |  |  |  |  |  |  |  |  |  |  |
|  | **Unmethylation** | 1.42 |  | (1.20 | – | 1.68) |  | <0.001 | 1.48 |  | (1.24 | – | 1.75) |  | <0.001 | 1.48 |  | (1.19 | – | 1.83) |  | <0.001 |
|  | **Unknown** | 1.19 |  | (0.97 | – | 1.46) |  | 0.096 | 1.10 |  | (0.90 | – | 1.36) |  | 0.347 | 1.09 |  | (0.84 | – | 1.42) |  | 0.504 |
| **Age** | **≥ 66** | [reference] |  |  |  |  |  |  |  |  |  |  |  |  |  |  |  |  |  |  |  |  |
|  | **≤ 65** | 0.93 |  | (0.80 | – | 1.07) |  | 0.303 | 1.00 |  | (0.86 | – | 1.17) |  | 0.979 | 1.05 |  | (0.87 | – | 1.26) |  | 0.619 |
| **KPS** | **≥ 90** | [reference] |  |  |  |  |  |  |  |  |  |  |  |  |  |  |  |  |  |  |  |  |
|  | **70–90** | 1.69 |  | (1.40 | – | 2.04) |  | <0.001 | 1.64 |  | (1.36 | – | 1.98) |  | <0.001 | 1.60 |  | (1.28 | – | 1.99) |  | <0.001 |
|  | **< 70** | 1.69 |  | (1.39 | – | 2.06) |  | <0.001 | 1.69 |  | (1.38 | – | 2.06) |  | <0.001 | 1.54 |  | (1.22 | – | 1.95) |  | <0.001 |
|  |  |  |  |  |  |  |  |  |  |  |  |  |  |  |  |  |  |  |  |  |  |  |
|  |  |  |  |  |  |  |  |  |  |  |  |  |  |  |  |  |  |  |  |  |  |  |
| *Adjusted for resection rate (GTR, non-GTR, biopsy), MGMT (methylation, unmethylation, unknown), age (≤ 65, ≥ 66), and KPS score (≥ 90, 70–90, < 70) | | | | | | | | | | | | | | | | | | | | | | |
| HR, hazard ratio |  |  |  |  |  |  |  |  |  |  |  |  |  |  |  |  |  |  |  |  |  |  |
| CI, confidence interval | |  |  |  |  |  |  |  |  |  |  |  |  |  |  |  |  |  |  |  |  |  |
| ASM, antiseizure medication | |  |  |  |  |  |  |  |  |  |  |  |  |  |  |  |  |  |  |  |  |  |
| PER, perampanel | |  |  |  |  |  |  |  |  |  |  |  |  |  |  |  |  |  |  |  |  |  |
| LEV, levetiracetam | |  |  |  |  |  |  |  |  |  |  |  |  |  |  |  |  |  |  |  |  |  |
| LCM, lacosamide | |  |  |  |  |  |  |  |  |  |  |  |  |  |  |  |  |  |  |  |  |  |
| GTR, gross total resection | |  |  |  |  |  |  |  |  |  |  |  |  |  |  |  |  |  |  |  |  |  |
| MGMT, O-6-methylguanine-DNAmethyltransferase | | | | | | | | |  |  |  |  |  |  |  |  |  |  |  |  |  |  |
| KPS, Karnofsky Performance Status | | |  |  |  |  |  |  |  |  |  |  |  |  |  |  |  |  |  |  |  |  |
