# Supplementary material for: Role of antiseizure medication in postoperative late seizure and survival among patients with newly diagnosed glioblastoma: A multicenter retrospective study
Source: Neurooncol Adv. 2026 Aug 7;8(1):vdag205. doi: 10.1093/noajnl/vdag205 (PMC13493057; doi:10.1093/noajnl/vdag205)
Supplement: Supplementary file 1 [file vdag205_supplementary_data.zip › Supplementary Table S7.docx]

| **Supplementary Table S7.** Hazard ratios for overall survival by antiseizure medication. | | | | | | | | | | | | | |  |  |  |  |  |  |  |  |  |
| --- | --- | --- | --- | --- | --- | --- | --- | --- | --- | --- | --- | --- | --- | --- | --- | --- | --- | --- | --- | --- | --- | --- |
|  |  |  |  |  |  |  |  |  |  |  |  |  |  |  |  |  |  |  |  |  |  |  |
|  |  |  |  |  |  |  |  |  |  |  |  |  |  |  |  |  |  |  |  |  |  |  |
|  |  | **Unadjusted** | | | | | | | **Multivariable Cox PH model*** | | | | | | | **Overlap weighting** | | | | | | |
|  |  | HR |  | 95% CI | | |  | p value | HR |  | 95% CI | | |  | p value | HR |  | 95% CI | | |  | p value |
|  |  |  |  |  |  |  |  |  |  |  |  |  |  |  |  |  |  |  |  |  |  |  |
| **ASM** | **No medication** | [reference] |  |  |  |  |  |  |  |  |  |  |  |  |  |  |  |  |  |  |  |  |
|  | **LEV** | 0.86 |  | (0.71 | – | 1.05) |  | 0.130 | 0.93 |  | (0.76 | – | 1.14) |  | 0.485 | 0.95 |  | (0.75 | – | 1.21) |  | 0.686 |
|  | **LCM** | 0.97 |  | (0.74 | – | 1.27) |  | 0.822 | 1.17 |  | (0.89 | – | 1.54) |  | 0.250 | 1.23 |  | (0.92 | – | 1.64) |  | 0.161 |
|  | **PER** | 0.83 |  | (0.64 | – | 1.06) |  | 0.132 | 1.05 |  | (0.81 | – | 1.35) |  | 0.722 | 1.05 |  | (0.79 | – | 1.38) |  | 0.752 |
| **Resection rate** | **GTR** | [reference] |  |  |  |  |  |  |  |  |  |  |  |  |  |  |  |  |  |  |  |  |
|  | **Non-GTR** | 2.03 |  | (1.69 | – | 2.45) |  | <0.001 | 1.90 |  | (1.57 | – | 2.30) |  | <0.001 | 2.02 |  | (1.63 | – | 2.51) |  | <0.001 |
|  | **Biopsy** | 2.03 |  | (1.61 | – | 2.56) |  | <0.001 | 1.80 |  | (1.41 | – | 2.30) |  | <0.001 | 1.55 |  | (1.06 | – | 2.27) |  | 0.025 |
| **MGMT** | **Methylation** | [reference] |  |  |  |  |  |  |  |  |  |  |  |  |  |  |  |  |  |  |  |  |
|  | **Unmethylation** | 1.46 |  | (1.20 | – | 1.77) |  | <0.001 | 1.59 |  | (1.30 | – | 1.94) |  | <0.001 | 1.54 |  | (1.2 | – | 1.98) |  | 0.001 |
|  | **Unknown** | 1.61 |  | (1.29 | – | 2.01) |  | <0.001 | 1.42 |  | (1.14 | – | 1.78) |  | 0.002 | 1.35 |  | (1.01 | – | 1.79) |  | 0.040 |
| **Age** | **≥ 66** | [reference] |  |  |  |  |  |  |  |  |  |  |  |  |  |  |  |  |  |  |  |  |
|  | **≤ 65** | 0.60 |  | (0.51 | – | 0.72) |  | <0.001 | 0.69 |  | (0.57 | – | 0.82) |  | <0.001 | 0.65 |  | (0.52 | – | 0.81) |  | <0.001 |
| **KPS** | **≥ 90** | [reference] |  |  |  |  |  |  |  |  |  |  |  |  |  |  |  |  |  |  |  |  |
|  | **70–90** | 1.80 |  | (1.43 | – | 2.25) |  | <0.001 | 1.66 |  | (1.32 | – | 2.09) |  | <0.001 | 1.77 |  | (1.35 | – | 2.30) |  | <0.001 |
|  | **< 70** | 2.29 |  | (1.83 | – | 2.88) |  | <0.001 | 2.01 |  | (1.59 | – | 2.53) |  | <0.001 | 1.91 |  | (1.45 | – | 2.51) |  | <0.001 |
|  |  |  |  |  |  |  |  |  |  |  |  |  |  |  |  |  |  |  |  |  |  |  |
|  |  |  |  |  |  |  |  |  |  |  |  |  |  |  |  |  |  |  |  |  |  |  |
| *Adjusted for resection rate (GTR, non-GTR, biopsy), MGMT (methylation, unmethylation, unknown), age (≤ 65, ≥ 66), and KPS score (≥ 90, 70–90, < 70) | | | | | | | | | | | | | | | | | | | | | | |
| HR, hazard ratio |  |  |  |  |  |  |  |  |  |  |  |  |  |  |  |  |  |  |  |  |  |  |
| CI, confidence interval | |  |  |  |  |  |  |  |  |  |  |  |  |  |  |  |  |  |  |  |  |  |
| ASM, antiseizure medication | |  |  |  |  |  |  |  |  |  |  |  |  |  |  |  |  |  |  |  |  |  |
| PER, perampanel | |  |  |  |  |  |  |  |  |  |  |  |  |  |  |  |  |  |  |  |  |  |
| LEV, levetiracetam | |  |  |  |  |  |  |  |  |  |  |  |  |  |  |  |  |  |  |  |  |  |
| LCM, lacosamide | |  |  |  |  |  |  |  |  |  |  |  |  |  |  |  |  |  |  |  |  |  |
| GTR, gross total resection | |  |  |  |  |  |  |  |  |  |  |  |  |  |  |  |  |  |  |  |  |  |
| MGMT, O-6-methylguanine-DNAmethyltransferase | | | | | |  |  |  |  |  |  |  |  |  |  |  |  |  |  |  |  |  |
| KPS, Karnofsky Performance Status | |  |  |  |  |  |  |  |  |  |  |  |  |  |  |  |  |  |  |  |  |  |
